# Improving seasonal forecasts of air temperature using a genetic algorithm

**DOI:** 10.1038/s41598-019-49281-z

**Published:** 2019-09-04

**Authors:** J. V. Ratnam, H. A. Dijkstra, Takeshi Doi, Yushi Morioka, Masami Nonaka, Swadhin K. Behera

**Affiliations:** 1 Application Laboratory, Japan Agency for Marine-Earth Science and Technology, Yokohama, Japan; 2 Institute for Marine and Atmospheric research, Utrecht university, Utrecht, The Netherlands

**Keywords:** Atmospheric dynamics, Environmental impact

## Abstract

Seasonal forecasts of air-temperature generated by numerical models provide guidance to the planners and to the society as a whole. However, generating accurate seasonal forecasts is challenging mainly due to the stochastic nature of the atmospheric internal variability. Therefore, an array of ensemble members is often used to capture the prediction signals. With large spread in the prediction plumes, it becomes important to employ techniques to reduce the effects of unrealistic members. One such technique is to create a weighted average of the ensemble members of seasonal forecasts. In this study, we applied a machine learning technique, viz. a genetic algorithm, to derive optimum weights for the 24-ensemble members of the coupled general circulation model; the Scale Interaction Experiment-Frontier research center for global change version 2 (SINTEX-F2) boreal summer forecasts. Our analysis showed the technique to have significantly improved the 2m-air temperature anomalies over several regions of South America, North America, Australia and Russia compared to the unweighted ensemble mean. The spatial distribution of air temperature anomalies is improved by the GA technique leading to better representation of anomalies in the predictions. Hence, machine learning techniques could help in improving the regional air temperature forecasts over the mid- and high-latitude regions where the model skills are relatively modest.

## Introduction

Lots of efforts are being made to understand the effects of climate change and to mitigate its consequences. However, there is also an urgent need to improve the skill of forecasting of climate at seasonal to interannual time scales, at least few months in advance. Successful seasonal forecasting of climate and extreme events would benefit planners and society as a whole. An ensemble of forecasts is often generated by varying initial conditions and also by varying parameters in the numerical models to increase the skill of seasonal forecasts. The ensemble of forecasts is then averaged to get an ensemble mean seasonal forecast. Figs S1–S3 show examples of the spatial distribution of the anomaly correlation coefficient (ACC) of the 24-members of the Scale Interaction Experiment-Frontier research center for global change version 2 (SINTEX-F2)^1,2^ forecast 2m-air temperature anomalies with respect to the Climate Research Unit (CRU)^3^ observed anomalies for the respective months from May initial conditions (see methods). The ACC values are for the forecasts of SINTEX-F2 for the months of June, July and August from May initial conditions. The figures highlight the spread in the ACC values among the members of the ensemble, with regions of negative and positive ACC values overlapping over some regions. The spread in the ACC values is prominent over sub-tropical to high latitude regions, due to large atmospheric internal variability not determined by the slowly varying boundary conditions such as sea surface temperature (SST). Clearly, a simple ensemble average created by adding up all the members, some of which are out of phase from each other, would annihilate the magnitude and phase of the anomalies thereby reducing the skill of the model forecasts.

This motivated us to look for techniques to generate better weighed ensemble means in which more weights are given to ensemble members, which predict more accurately the phase and magnitude of the observed anomalies. A technique that is widely used to average the ensemble forecasts from a number of models to generate a single forecast is called the Multi Model Ensemble (MME) forecast^4–6^. In generating the MME forecasts, individual models are evaluated separately for their skills and weights are given to the models based on their individual skills. However, the challenge in generating an MME forecast is to quantify the model skill and derive weights for each model^7^.

Multiple linear regression of the model forecasts with an observed field and then using least-square techniques to minimize the difference between model and observed field, is one of the techniques used for deriving the model weights^4,5^ for generating MME forecast. The model weights can also be derived non-linearly using the artificial intelligence techniques such as artificial neural network (ANN)^8,9^ and genetic algorithm (GA)^6^. The GAs are a class of search techniques that imitate the biological processes of selection, inheritance and variation^10^. They can also be used to solve optimization problems^10^. MME forecasts generated using the weights from a GA have been demonstrated to be more skillful compared to the method of giving equal weights to models and generating an ensemble mean^6^.

Although the technique of weighted average is often used for generating MME forecasts, in this study we apply a GA, PIKAIA^10^, to generate a weighted ensemble mean of an ensemble of 24 members from a single seasonal forecast model SINTEX-F2 due to the efficiency and ease of applying the technique. The ensemble members of SINTEX-F2 forecasts are generated by varying the physical schemes in the model, by varying the sea surface temperature and by including the sub-surface initialization techniques. The detrended anomalies of the boreal summer (June-August) monthly 2 m-air temperature forecasts from 1^st^ May initial conditions, for the period 1983–2015, are used in the study. As is well known, the skill of the forecasts of the numerical models reduces with higher lead times. Hence, we chose the retrospective forecasts from 1^st^ May initial conditions to keep the lead time to 1 month i.e. for considering the predictions of June-August season. A GA (see methods) technique is applied to derive weighted averaged SINTEX-F2 forecasts. The ensemble average generated by giving equal weights to all the members is hereafter referred to as SINTEX-F2 and the weighted ensemble average with the weights generated from the GA is referred to as SINTEX-F2ga.

## Results

### Improvements in June air-temperature forecast

**Figure 1:**
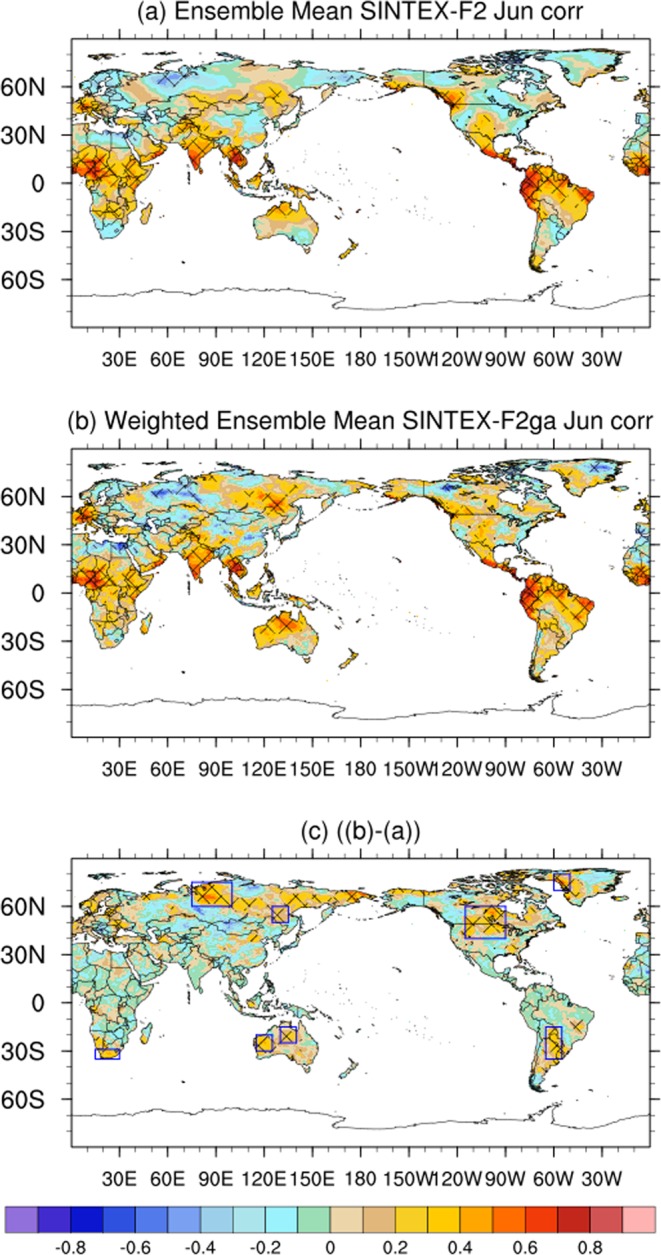
(**a**) Spatial distribution of ACC values of SINTEX-F2 2m-air temperature anomalies with respect to CRU anomalies for the month of June. (**b**) same as (**a**) but for SINTEX-F2ga (**c**) Difference in ACC values between SINTEX-F2ga and SINTEX-F2. The regions of significant (at 90% using Student’s 2-tailed t-test) ACC values are hashed. The rectangular boxes in (**c**) are the regions of significant differences in ACC values between SINTEX-F2ga and SINTEX-F2. The figure was prepared using The NCAR Command Language (version 6.4.0) [Software]. (2017). Boulder, Colorado: UCAR/NCAR/CISL/TDD. http://dx.doi.org/10.5065/D6WD3XH5.

The ACC of the ensemble mean of the SINTEX-F2 June forecast is significant and positive over most of the tropical regions (Fig. 1a) with values higher than 0.4 over west Africa, southern parts of India, most of the south eastern Asian countries, Northern Territory of Australia, Mexico, and tropical South America, the regions where most of the members of the SINTEX-F2 ensemble agree on the phase of the anomalies (Fig. S1). The regions of statistically significant positive ACC values over sub-tropical and high latitudes are scattered and are located over west Canada, eastern parts of Russia and over west Europe (Fig. 1a). The climate of the tropical regions is mostly determined by the slowly varying sea surface temperature (SST) and the atmospheric rising and sinking motions associated with it, whereas the climate of higher-latitudes is determined by the high-frequency atmospheric processes. Thus, the spatial distribution of the ACC values of 2m-air temperature in the tropical regions can be partly explained by analyzing the ACC values of the forecasted SST anomalies and the spatial distribution of 200 hPa streamfunction.

The forecasted SST anomalies by SINTEX-F2 for the month of June have significantly positive and high ACC values exceeding 0.6 over the equatorial regions (Fig. S4a). The high skill of the SINTEX-F2 model in forecasting the equatorial SST anomalies leads to a realistic representation of rising and sinking motions in the atmosphere or in other words a realistic simulation of the Walker circulation. The 200 hPa velocity potential, a proxy of Walker circulation, for the June forecast (S5a) has significant ACC values corresponding to the regions of positive ACC values of SST (Fig. S4a) and of 2m-air temperature anomalies (Fig. 1a). To explain the positive ACC values of the 2m-air temperature anomalies over the scattered regions in the higher latitudes, we plotted the ACC of the SINTEX-F2 200 hPa streamfunction anomalies with ERA-Interim estimated streamfunction anomalies for all the 24 members (Fig. S6). Comparison of Figs S1, S6 reveals that the SINTEX-F2 members which could realistically forecast the 200 hPa streamfunction anomalies in the higher latitudes, due to barotropicity of the high-latitude atmosphere could also forecast a correct phase of the temperature anomalies over the region.

**Figure 2:**
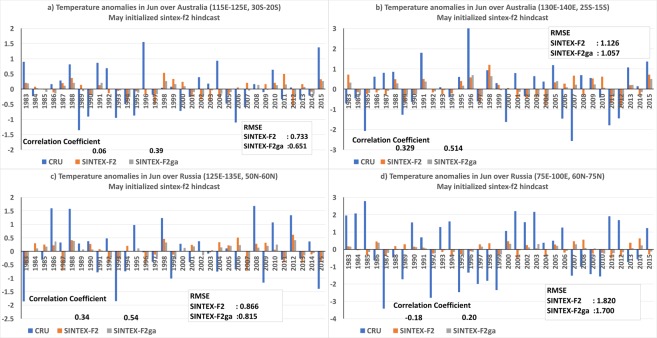
Interannual variation of 2m-air temperature anomalies over (**a**) west Australia, (**b**) northern Territory, Australia, (**c**) east Russia and (**d**) north Russia of CRU, SINTEX-F2 and SINTEX-F2ga for the month of June. The panels were prepared using Microsoft EXCEL 2016 and merged with ImageMagick software (version 6.7.2-7) (https://imagemagick.org/).

Interestingly, the weighted ensemble average of the June forecasts, SINTEX-F2ga, enlarged the regions of significant positive ACC values over Australia, with the significant ACC values covering the regions of Western Australia (115°E–125°E; 20°S–30°S), Northern Territory (130°E–140°E; 15°S–25°S) (Fig. 1b). The region of significant positive ACC values is also enhanced over eastern parts of Russia (125°E–135°E; 50°N–60°N) and northern Russia (75°E–100°E; 60°N–75°N) (Fig. 1b). All the above regions are shown as rectangular boxes in Fig. 2c. The differences in the ACC values between SINTEX-F2ga and SINTEX-F2 are also significant over these regions of significantly improved ACC values (Fig. 2c). The improvement in ACC values due to weighted ensemble is also evident over South Africa (15°E–30°E; 29°S–35°S), South America (295°E–205°E; 15°S–35°S), USA and Canada (245°E–270°E; 40°N–60°N), Greenland (300°E–310°E; 70°N–80°N). Although the difference in the ACC values is significant over these regions (Fig. 2c), the ACC values are not statistically significant in SINTEX-F2ga (Fig. 2b). The weighted ensemble average, on the downside, reduces the ACC values over the tropical regions and over west Canada (Fig. 2b) but those reductions are not statistically significant (Fig. 2c).

We plotted the weights generated by the GA (Fig. S7) for all the 24 members (Fig. S6) to investigate the source of the ACC improvements in higher latitudes. Interestingly, the spatial distribution of the higher weights (larger than 0.8), assigned by the GA corresponds to the regions of positive ACC values of 2m-air temperature anomalies in June (Fig. S1). The GA assigned low weights (Fig. S7) to the regions with negative ACC values for 2m-air temperature forecasts (Fig. S1). An ensemble average, after applying these weights, reduces the effect of out-of-phase anomalies and thus leads to an improvement of the 2m-air temperature forecast in June over the higher-latitudes. Over the tropical regions, the regions of high ACC values (Fig. 1a) (where most of the members agree on the phase of the anomalies (Fig. S1)), the weighted average reduces the magnitudes of the anomalies thus reducing the ACC values by about 0.1–0.2 (Fig. 1c), which are not statistically significant (Fig. 1c).

The ACC values of the weighted ensemble mean of June 2m-air temperature anomalies using the three-fold cross validation technique is shown in Fig. S8b,c. Comparing Fig. S8c and Fig. 1c, it is evident that the regions of significant improvement in ACC values over the higher latitudes are not sensitive to the choice of the training period. However, the magnitude of ACC values is higher using the jackknife method, of leave-one out, partly due to the size of the dataset with only 33-years.

We examined the interannual variation of the 2m-temperature anomalies to investigate the source of improvements in the weighted ensemble mean SINTEX-F2ga June forecast. Figure 2 shows the area-averaged June 2m-air temperatures anomalies over the regions of Australia and Russia, which have statistically significant ACC values in SINTEX-F2ga (Fig. 1b,c). The ACC values over the Western Australia (115°E–125°E; 20°S–30°S) improve from 0.06 in SINTEX-F2 to 0.39 in the weighted ensemble mean SINTEX-F2ga (Fig. 2a) and the root mean square error (RMSE) is reduced from 0.73 °C to 0.65 °C. The weighted ensemble average corrects the phase of the temperature anomalies in the June of 1989, 1996, 2007 and 2009 (Fig. 2a) and improves the amplitude of the anomalies in several years (Fig. 2a) resulting in the improvement in the ACC value and a reduction of RMSE. The spatial distribution of the June 2m-air temperature anomalies in the years 1989, 1996, 2007 and 2009 (Fig. S9), in which the phase of the anomalies over Western Australia is corrected in the weighted mean, illustrates the benefits of using the weighted mean compared to the unweighted ensemble mean. In all the years 1989, 2007 and 2009 when the CRU estimated air temperature anomalies were negative over the Western Australia (Fig. S9a,g,j), SINTEX-F2 forecasts positive anomalies (Fig. S9b,h,k). The weights generated by the GA when applied to the ensemble members is able to correct the phase of the anomalies over Western Australia (Fig. S9c,i,j). Similarly, the phase of the anomalies in the June of 1996 (Fig. S9d,e,f) is also improved by the weighted average.

Over Northern Territory (130°E–140°E; 25°S–15°S), the correlation coefficient is improved from 0.329 in the unweighted ensemble mean SINTEX-F2 to 0.51 in the weighted ensemble mean SINTEX-F2ga (Fig. 2b) and RMSE is reduced from 1.13 °C to 1.05 °C due to improvement in the magnitude of the anomalies. Similarly, the correlation coefficient increased from 0.34 to 0.54 and RMSE is reduced from 0.86 °C to 0.81 °C over eastern parts of Russia (125°E–135°E; 50°N–60°N) in SINTEX-F2ga compared to SINTEX-F2, due to improvement in the amplitude of the anomalies in the weighted ensemble mean (Fig. 2c). The correlation coefficients over northern Russia (75°E–100°E; 60°N–75°N) improved from −0.18 to 0.20 in SINTEX-F2ga mean compared to SINTEX-F2 mean (Fig. 2d).

The RMSE and ACC values of SINTEX-F2 and SINTEX-F2ga over different regions for June forecasts is shown in Table 1. From the table it can be seen that the area averaged 2m-air temperature anomalies over USA and Canada have improved ACC values from −0.13 to 0.16 and a reduction of RMSE from 1.15 °C to 1.07 °C in SINTEX-F2ga mean compared to SINTEX-F2 mean. The ACC values over Greenland increased from −0.13 to 0.239 and RMSE values decreased from 0.88 °C to 0.78 °C; over South Africa the correlation coefficient improved from −0.19 to 0.12 and RMSE reduced from 0.68 °C to 0.57 °C; over South America (295°E–305°E; 35°S–15°S) ACC improved from −0.063 to 0.245 and RMSE is reduced from 1.24 °C to 1.13 °C in the weighted ensemble mean compared to the unweighted ensemble mean.

**Table 1:** ACC and RMSE values of SINTEX-F2 and SINTEX-F2ga over different regions in the June forecast.

June	SINTEX-F2		SINTEX-F2ga	
	ACC	RMSE	ACC	RMSE
west Australia (115°E–125°E; 20°S–30°S)	0.06	0.73	0.39	0.65
Northern Territory (130°E–140°E; 25°S–15°S)	0.33	1.13	0.51	1.05
east Russia (125°E–135°E; 50°N–60°N)	0.34	0.86	0.54	0.81
north Russia (75°E–100°E; 60°N–75°N)	−0.18	1.82	0.20	1.70
USA and Canada (245°E–270°E; 40°N–60°N)	−0.13	1.15	0.16	1.07
Greenland (300°E–310°E; 70°N–80°N)	−0.13	0.88	0.24	0.78
South Africa (15°E–30°E; 29°S–35°S)	−0.19	0.68	0.12	0.57
South America (295°E–205°E; 15°S–35°S)	−0.06	1.24	0.245	1.13

### Improvements in July air-temperature forecast

**Figure 3:**
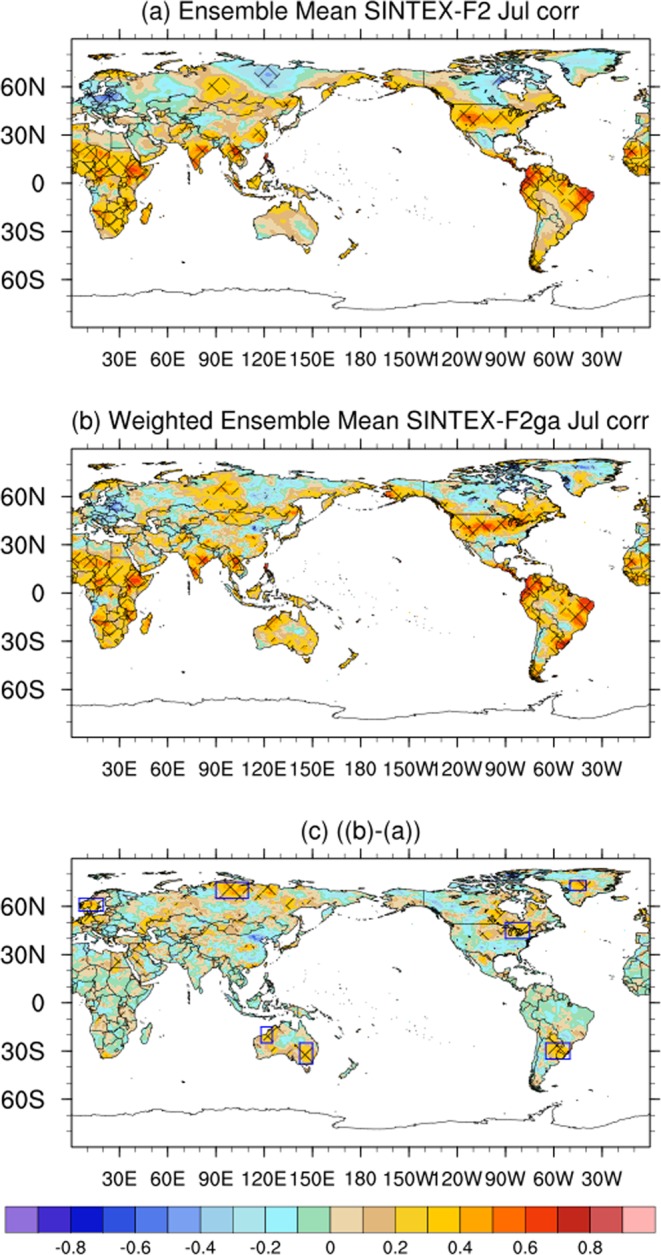
Same as Fig. 1. but for the month of July. The figure was prepared using The NCAR Command Language (version 6.4.0) [Software]. (2017). Boulder, Colorado: UCAR/NCAR/CISL/TDD. http://dx.doi.org/10.5065/D6WD3XH5.

The ensemble average of the SINTEX-F2 forecast July 2m-air temperatures anomalies have significant positive correlation coefficients over most parts of the tropical regions except over Mexico and South Arabia (Fig. 3a). Over the sub-tropics and higher latitudes, the 2m-air temperatures anomalies have positive and significant correlations only over USA, and parts of Russia (Fig. 3a). The ACC values over USA, Australia and South America, north Russia, Greenland and west Europe are improved by applying the weighted average (Fig. 3b,c). Over northern parts of Western Australia (118°E–125°E; 15°S–25°S), over sub-tropical South America (295°E–310°E; 25°S–35°S) and over sub-tropical USA (270°E–285°E; 40°N–50°N) the ACC values become significant in the SINTEX-F2ga mean (Fig. 3b). The differences in ACC values between SINTEX-F2ga and SINTEX-F2 are also significant over those regions (Fig. 3c). The differences in ACC values over north Russia (90°E–110°E; 65°N–75°N), Greenland (310°E–320°E; 70°N–76°N), west Europe (5°E–20°E; 57°N–65°N), and over southeast Australia (142°E–150°E; 25°S–38°S) between SINTEX-F2ga mean and SINTEX-F2 mean are statistically significant (Fig. 3c). However, the ACC values are not statistically significant in both SINTEX-F2 and SINTEX-F2ga mean over these regions (Fig. 3a,b). The high positive ACC values of SST (Fig. S4b) along with the positive ACC values of 200 hPa velocity potential (Fig. S5b) explain the positive ACC values of 2m-air temperature anomalies in the tropical regions in July (Fig. 3a). The ACC of 2m-air temperature anomalies in the higher latitudes is well explained by the ACC values of 200 hPa streamfunction anomalies in July (Fig. S10). The regions of positive ACC values of streamfunction anomalies correspond to the regions of positive ACC values of 2m-air temperature anomalies and vice-versa. The spatial distribution of the weights generated by the GA for the July forecast (Fig. S11) demonstrates the ability of the GA to assign higher weights to the ensemble members with regions corresponding to higher ACC values of 2m-air temperature anomalies and lower values to members with regions with negative ACC values in the higher latitudes. The weighted average created by applying these weights improved the 2m-air temperature forecasts over high latitudes.

Comparing Fig. S12b,c with Fig. 3b,c, it is evident that the three-fold cross validation of the July forecasts (similar to the June forecasts) does not affect the regions of significant improvement in the ACC values over the high-latitudes but the magnitudes of the ACC values are reduced.

**Figure 4:**
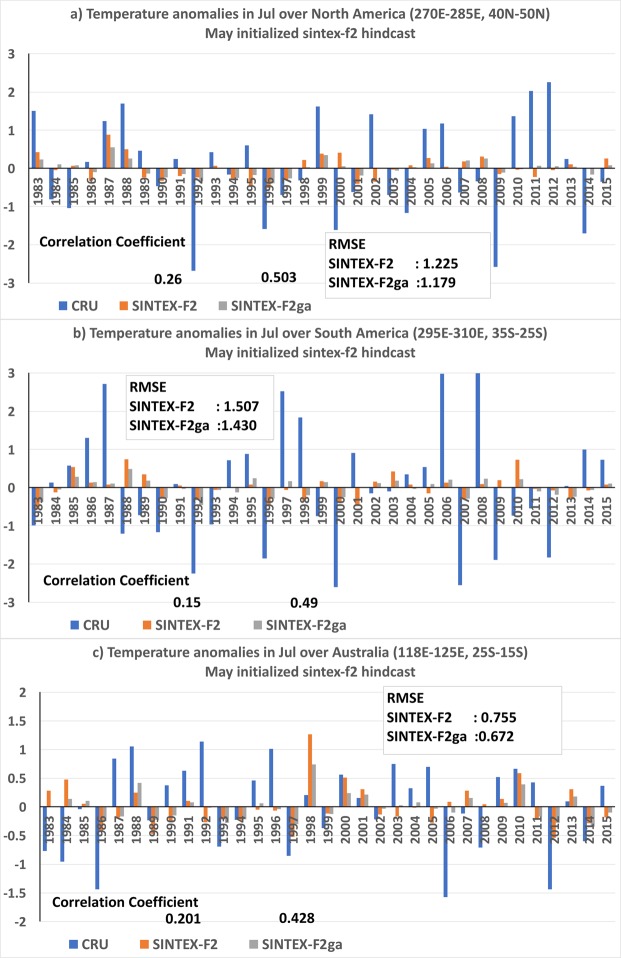
Interannual variation of 2m-air temperature anomalies over (**a**) USA (**b**) South America and (**c**) west Australia of CRU, SINTEX-F2 and SINTEX-F2ga for the month of July. The panels were prepared using Microsoft EXCEL 2016 and merged with ImageMagick software (version 6.7.2-7) (https://imagemagick.org/).

The interannual variation of July temperature anomalies over USA (270°E–285°E; 40°N–50°N) has a correlation of 0.26 in the unweighted ensemble mean which is enhanced to 0.503 in the weighted ensemble mean (Fig. 4a). Examining Fig. 4a, we find that the enhancement of the correlation coefficient is due correction of phase of temperature anomalies in the years 2011 and 2012 and also due to improvement in the magnitude of the anomalies over many years. The RMSE over USA is reduced from 1.22 °C to 1.17 °C in SINTEX-F2ga compared to SINTEX-F2. The spatial distribution of anomalies in July of 2011 and 2012 (Fig. S13) clearly brings out the improvement by the weighted mean over the USA region (rectangular box). In both the years USA experienced anomalously high July temperatures (Fig. S13a,d). The SINTEX-F2, though could forecast the anomalous high temperatures over USA in those years, the spatial extent of the high temperatures was limited to southern parts of the USA (Fig. S13b,e). The weighted mean could correct the temperature anomalies over the USA region (box in Fig. S13c) in July of 2011 and thereby improve the phase of the anomalies over the region. In July of 2012, the SINTEX-F2ga mean shows reduction of negative anomalies over the USA region (Fig. S13f) and thereby improve the anomalies.

Over sub-tropical South America, the ACC values is increased from 0.15 to 0.49 and RMSE is reduced from 1.50 to 1.43 in SINTEX-F2ga mean compared to SINTEX-F2 mean (Fig. 4b). The weighted average corrects the phase of the anomalies in July of 1997, 2005 and improved the magnitude of the anomalies over many years (Fig. 4b). The spatial distribution of the air-temperature anomalies in July of 1997 and 2005 (Fig. S14) illustrates the phase correction by the weighted mean over the sub-tropical South America region (rectangular box in Fig. S14). In both July 1997 and 2005, the region experienced anomalously high temperatures (Figs S14a,d). SINTEX-F2 forecasts had bias in the spatial extent of the positive temperature anomalies over the region (Fig. S14b,e) which was corrected in the SINTEX-F2ga temperature anomalies (Fig. S14c,f).

Over northern parts of Western Australia (118°E–125°E; 25°S–15°S) the ACC values are improved from 0.20 to 0.43 (Fig. 4c) and the RMSE is reduced from 0.75 °C to 0.67 °C in the SINTEX-F2ga mean compared to SINTEX-F2 mean. The correction of phase of the air-temperature anomalies is in the July of 1995, 2003, 2004 and 2006 and amplitude of the anomalies is improved in several years in the SINTEX-F2ga mean compared to SINTEX-F2 mean (Fig. 4c). Figure S15 shows the spatial distribution of the air-temperature anomalies over Australia in the July of 1995, 2003, 2004 and 2006, the years in which the phase of the anomalies is improved over the northern parts of Western Australia. It is clear from Fig. S15 that the weighted mean has the advantage over the simple weighted mean in forecasting the temperatures over the northern parts of Western Australia in July.

Table 2 shows the RMSE and ACC values over different regions in the month of July. From the table it is evident that the improvement in ACC values are modest over the southeast Australia, with values of −0.08 in SINTEX-F2 mean and 0.20 in SINTEX-F2ga mean; over north Russia with values of −0.09 in SINTEX-F2 mean and 0.24 in SINTEX-F2ga mean; over Greenland from −0.16 to 0.21 and over west Europe from −0.05 to 0.20 from SINTEX-F2 mean to SINTEX-F2ga mean.

**Table 2:** ACC and RMSE values of SINTEX-F2 and SINTEX-F2ga over different regions in the July forecast.

July	SINTEX-F2		SINTEX-F2ga	
	ACC	RMSE	ACC	RMSE
USA (270°E–285°E; 40°N–50°N)	0.26	1.22	0.50	1.17
South America (295°E–310°E; 25°S–35°S)	0.15	1.50	0.49	1.43
west Australia (118°E–125°E; 25°S–15°S)	0.20	0.75	0.43	0.67
southeast Australia (142°E–150°E; 25°S–38°S)	−0.08	0.68	0.20	0.58
north Russia (90°E–110°E;65°N–75°N)	−0.09	1.33	0.24	1.16
Greenland (310°E–320°E;70°N–76°N)	−0.16	0.59	0.21	0.46
west Europe (5°E–20°E;57°N–65°N)	−0.05	1.28	0.20	1.16

### Improvements in August air-temperature forecast

**Figure 5:**
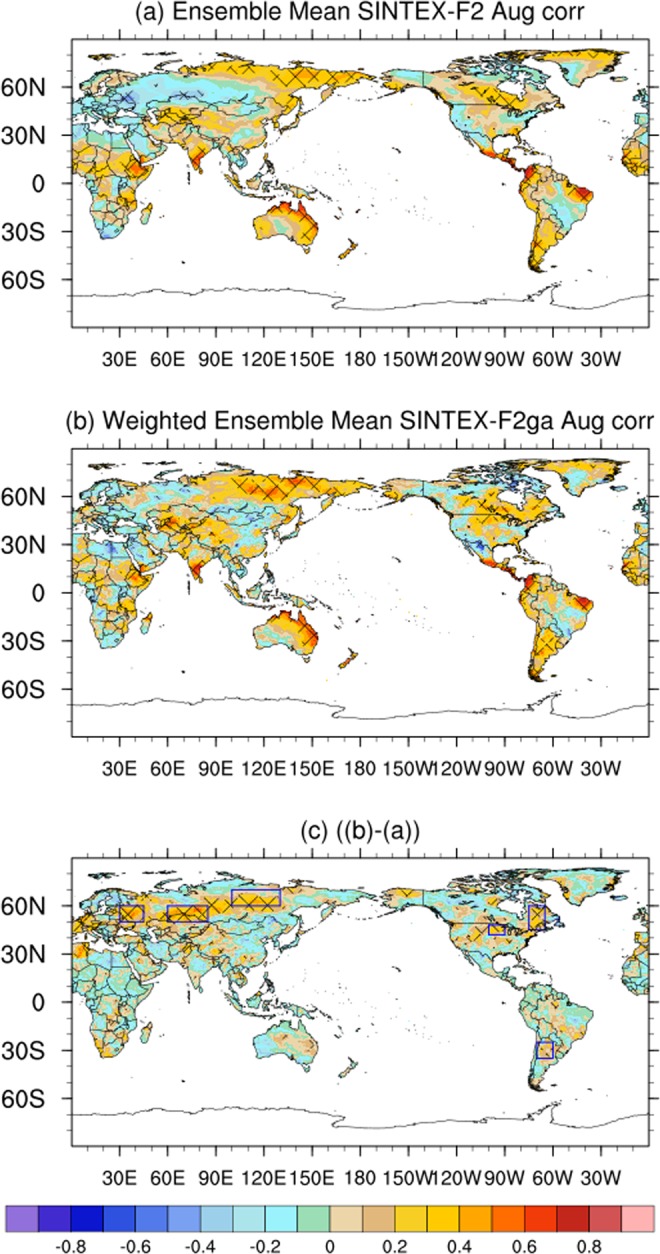
Same as Fig. 1. but for the month of August. The figure was prepared using The NCAR Command Language (version 6.4.0) [Software]. (2017). Boulder, Colorado: UCAR/NCAR/CISL/TDD. http://dx.doi.org/10.5065/D6WD3XH5.

The ACC values of the SINTEX-F2 mean August forecast are positive and significant over parts of south India, east Africa, Mexico, parts of Australia, east Russia and parts of Canada (Fig. 5a). The ACC values are enhanced and become statistically significant over sub-tropical South America, east Canada, and over parts of USA in the weighted mean SINTEX-F2ga (Fig. 5b). The differences in ACC values is positive and significant over parts of east Europe (30°E–45°E; 50°N–60°N), east Russia (100°E–130°E; 60°N–70°N), west Russia (60°E–85°E; 50°N–60°N), east Canada (285°E–295°E; 45°N–60°N), parts of USA (260°E–270°E;42°N–48°N, sub-tropical South America (290°E–300°E; 25°S–35°S) (Fig. 5c). The regions are shown as rectangular boxes in Fig. 5c. The ACC of SINTEX-F2 forecast SST anomalies is positive over most parts of the equatorial regions except over a small region near the west Pacific and over south Indian Ocean (Fig. S4c). The ACC of 200 hPa SINTEX-F2 forecast August velocity potential anomalies is high over the tropical regions, indicating the atmospheric response of rising and sinking motions in the tropical regions to be realistically forecasted by SINTEX-F2, thereby largely explaining the ACC values 2m-air temperature anomalies in the tropical regions (Fig. 5a). The ACC of 2m-air temperature anomalies in the higher latitudes is well explained by the ACC values of 200 hPa streamfunction anomalies in August (Fig. S16). The spatial distribution of the weights generated by the GA for the August forecast (Fig. S17) illustrates the ability of the genetic algorithm to assign weights realistically to the members of the SINTEX-F2 forecast. The genetic algorithm assigned higher weights to the regions corresponding to higher ACC values of 2m-air temperature anomalies and lower values to regions with negative ACC values in the higher latitudes. The weighted average created by applying these weights improved the 2m-air temperature forecasts over high-latitudes. Over the tropical regions, the ACC values are decreased by about 0.1 to 0.2, though the decreases are not statistically significant.

The weighted ensemble average of the anomalies of the SINTEX-F2 August forecast using the using the three-fold cross validation technique is shown in Fig. S18. Comparing Fig. S18b,c with Fig. 5b,c, it is clear that the three-fold cross validation has lower ACC values compared to the jackknife technique but the regions of significant improvement in the forecast over the high-latitudes are not changed.

**Figure 6:**
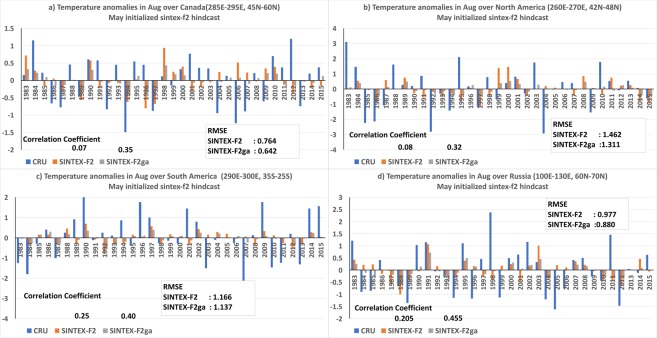
Interannual variation of 2m-air temperature anomalies over (**a**) Canada (**b**) USA and (**c**) South America and (**d**) Russia of CRU, SINTEX-F2 and SINTEX-F2ga for the month of August. The panels were prepared using Microsoft EXCEL 2016 and merged with ImageMagick software (version 6.7.2-7) (https://imagemagick.org/).

The interannual variation of the air-temperature anomalies over east Canada (285E–295E; 45N–60N) indicates an improvement in ACC values from 0.07 to 0.35 in the weighted mean SINTEX-F2ga compared to SINTEX-F2 mean (Fig. 6a). The RMSE is reduced from 0.76 °C in SINTEX-F2 mean to 0.64 °C in the SINTEX-F2ga mean. The phase of the anomalies is corrected in SINTEX-F2ga in the August of 1985, 1986, 2004, 2005, 2007, 2008, 2013 and 2015 (Fig. 6a). Over parts of USA (260°E–270°E; 42°N–48°N) the correlation of the anomalies increases from 0.08 to 0.32 and RMSE is reduced from 1.46 °C to 1.31 °C in the SINTEX-F2ga mean compared to SINTEX-F2 mean (Fig. 6b). The amplitude of the anomalies is improved in most of the years and phase is corrected in the August of 2010 (Fig. S19) in SINTEX-F2ga. The ACC over sub-tropical South America (290°E–300°E; 35°S–25°S) improved from 0.25 to 0.40 in the SINTEX-F2ga mean compared to SINTEX-F2 mean (Fig. 6c) and the RMSE is reduced from 1.16 °C in SINTEX-F2 mean to 1.13 °C in the SINTEX-F2ga mean. The ACC (RMSE) over the east Russia is 0.20 (0.97 °C) in the SINTEX-F2 mean which is increased (decreased) to 0.45 (0.88 °C) in the SINTEX-F2ga mean (Fig. 6d). Correction of phase of the anomalies is seen in the years 1984, 1985, 1990, 1992, 2001 and amplitude of the anomalies are improved in many years in the SINTEX-F2ga mean compared to the SINTEX-F2 mean (Fig. 6d) over the region.

Over west Russia the ACC improved from a negative value of −0.345 to 0.016 and RMSE decreased from 1.28 °C to 1.15 °C (Table 3) and over east Europe the ACC improved from −0.39 to −0.04 with reduction of RMSE from 1.23 °C to 1.04 °C in SINTEX-F2ga compared to SINTEX-F2 ensemble mean (Table 3).

**Table 3:** ACC and RMSE values of SINTEX-F2 and SINTEX-F2ga over different regions in the August forecast.

August	SINTEX-F2		SINTEX-F2ga	
	ACC	RMSE	ACC	RMSE
east Canada (285E–295E; 45N–60N)	0.07	0.76	0.35	0.64
USA (260°E–270°E; 42°N–48°N)	0.08	1.46	0.32	1.31
South America (290°E–300°E; 35°S–25°S)	0.25	1.16	0.40	1.13
east Russia (100°E–130°E; 60°N–70°N)	0.20	0.97	0.45	0.88
west Russia (60°E–85°E; 50°N–60°N),	−0.34	1.28	0.02	1.15
east Europe (30°E–45°E; 50°N–60°N)	−0.39	1.23	−0.04	1.04

## Discussion

In the present study we improved the 2m-air temperature anomalies of the one-month lead boreal summer seasonal forecast of SINTEX-F2 model by applying GA that is the weighted averaging technique. A 24-member ensemble of SINTEX-F2 forecasts initialized in May from 1983 to 2015 was used for the analysis. The GA generations used RMSE as the fitting function.

The spatial distribution of the ACC scores of June forecasts showed the SINTEX-F2 ensemble mean to have significant values over most of the tropical countries as well as over west Canada, eastern parts of Russia and over west Europe in the sub-tropical and high-latitude regions. The weighted ensemble average SINTEX-F2ga, by using the weights assigned by the genetic algorithm to the members of the SINTEX-F2 forecast, significantly improved the 2m-temperature anomalies over several regions of the globe. The regions over South Africa, South America, USA and Canada and Greenland had significantly improved ACC and reduced RMSE values. Analysis of the interannual variation of the 2m-air temperature anomalies and their spatial distribution indicated the improvement in the ACC scores and reduction of RMSE over the regions that are associated with realistic spatial representation of the anomalies and improvement in the amplitude of the anomalies in the SINTEX-F2ga ensemble mean.

Similarly, in July the regions of significantly improved ACC values and reduced RMSE values in the weighted ensemble mean SINTEX-F2ga are located over north Russia, Greenland, west Europe, and over southeast Australia. In August, east Canada, parts of USA, east Russia, west Russia, and east Europe showed improvements in ACC values in the SINTEX-F2ga. The improvements were due to better spatial distribution of the 2m-air temperature anomalies over the region in SINTEX-F2ga.

We also applied the GA technique to the 1^st^ November initialized SINTEX-F2 forecasts of boreal winter months of December to February, to test the sensitivity of the results to the season. The plots of the spatial distribution of the 2m-temperature anomalies are shown in Figs S20–S22. The plots clearly show improvement over the mid- and high-latitude regions by applying the GA technique to the SINTEX-F2 forecasts, though the regions of improvement are different between the boreal summer and winter forecasts. This indicates that the GA technique is an important tool to improve the 2m-air temperature forecasts over the mid- and high-latitudes, though some of the regions of improvements are season dependent.

The improvements in the phase and amplitude of the air-temperature anomalies is a significant step forward to improve the SINTEX-F2 forecasts. There are several techniques to improve the mean values of the forecasts^11^ but it is a challenge to improve the phase and amplitude of the anomalies of the forecasts^12^. This study shows that deriving hidden information inherent among the members of the SINTEX-F2 forecasts by applying a machine learning technique such as GA can improve the air-temperature anomalies significantly over the mid- and high-latitude regions. The numerical models due to the dominance of the noise in the forecasts over mid- and high-latitudes, have relatively modest skill compared to the forecasts over the tropical regions. Any improvement in the skill of the forecasts over mid- and high-latitudes is a step forward in the art of forecasting. The main contribution of this study is to demonstrate that applying the GA based machine learning technique is useful for improving the 2m-air temperature forecasts over mid- and high-latitudes.

## Methods

### SINTEX-F2 model forecasts and verification datasets

SINTEX-F2 seasonal forecasting model is an atmosphere-ocean coupled model with the atmospheric component at a horizontal resolution of approximately 1° × 1° (~100 km) and the oceanic component at 0.5° × 0.5° horizontal resolution. An ensemble of 24- member forecasts from the SINTEX-F2 model were used in the analysis. The ensemble was generated by varying the method of initialization of the Sea Surface Temperatures and the subsurface ocean observation, and also the physical scheme^1,2^. The retrospective forecasts were issued on May 1^st^ for all the years from 1983 to 2015, and covering the June-August period Monthly 2m-air temperature anomalies were generated from the forecasts with for all the years from 1983 to 2015.

The Climate Research Unit (CRU) estimated surface air temperature (CRU TS v.4.02)^3^ was used as the validation data set for validating the 2 m-air temperatures. The CRU dataset is at horizontal resolution of 0.5° × 0.5°. The CRU air-temperature anomalies were derived for the months of June-August for the years 1983 to 2015 with the average of air-temperature for the period 1983 to 2015 taken as the climatology. The SINTEX-F2 forecasts were linearly interpolated to the CRU grid. The CRU and SINTEX-F2 air-temperature anomalies were linearly detrended to remove the trend in the data. The SST forecast by the SINTEX-F2 is validated against the OIV2 estimated SST values. The 200 hpa ERA-Interim estimated zonal and meridional wind was used to derive the 200 hPa streamfunction. The SINTEX-F2 forecast 200 hPa streamfunction anomalies were compared against the ERA-interim estimated anomalies. The anomalies of all the variables were linearly detrended to remove trend in the data. The NCAR command language (NCL) version 6.4.0 was used to generate streamfunction and Climate Data Operators (CDO) version 1.8.0rc4 was used for detrending. The Student’s two-tailed t-test is used to test the significance (at 90%) of the anomaly correlations. The significance (at 90%) of difference in the anomaly correlations is tested following the methodology of Siegert *et al*.^13^.

### Genetic algorithm

The generic algorithm, PIKAIA^10^, developed at the High Altitude Observatory of National Center for Atmospheric Research, USA was used in our study to generate the weights for the members of the SINTEX-F2 forecasts. PIKAIA is a free software and can be downloaded from http://www.hao.ucar.edu/modeling/pikaia/pikaia.php. The model was set up to evolve over 1000 generations with cross over probability of 0.85 and with steady-state-replace-worst strategy. The strategy for mutation within the model was based on one-point adjustable rate based on fitness. The initial mutation rate was set to 0.01; with a bound between 0.001 and 0.25 over the model integration. It is found that slight changes to these values by varying the mutation rate between 0.005 or 0.015 did not affect the final results much.

All the members of the SINTEX-F2 model forecast monthly air-temperature anomalies are read by the program. At the first step, using a random number generator, random weights are assigned to the members. The RMSE is used as a fitness function in our study. The RMSE of the members with the random weights are evaluated. The PIKAIA tries to reduce the RMSE with each iteration by varying the weights to the members. This process of reducing the RMSE of the forecast is repeated till the model reaches a stable state and optimum weights for each member are obtained. The above process is applied to each grid point separately. We used the jackknife^14^ approach of leave-one-out to get the weights for the training period (32 years) and applied the weights to the verification year (1 year) for all the years from 1983 to 2015. We found that the model reaches a stable state after about 500 generations for all the grid points. We used the weights generated by the model after 1000 iterations as the weights for each model for each grid point to generate the weighted ensemble average. In addition, we also applied the 3-fold cross validation technique to check the sensitivity of the results to the choice of the training period and to check for the over-fitting. The period of 33 years (from 1983 to 2015) is divided into three folds after randomly rearranging the years without replacement. The random sample of years we used for the analysis is 2011, 1989, 1993, 1996, 1983, 1986, 2007, 2003, 2010, 1998, 1988, 1995, 1991, 2014, 2006, 1984, 1985, 1992, 1987, 1997, 2009, 2001, 1999, 2000, 1994, 2002, 2008, 2012, 2005, 2004, 2013, 1990, 2015. As the first fold we considered the first 22 years of the above sample for training and the last 11 years of the sample for verification. The second fold consisted of the last 22 years of the above sample for training and the first 11 years of the sample for verification. The third fold had 22 years from the first 11 and the last 11 years of the sample for training and the middle 11 years of the sample for verification.

## Supplementary information

Supplementary Figures
