# Supplementary material for: Improving seasonal forecasts of air temperature using a genetic algorithm
Source: Sci Rep. 2019 Sep 4;9:12781. doi: 10.1038/s41598-019-49281-z (PMC6726601; doi:10.1038/s41598-019-49281-z)
Supplement: Supplementary file 1 — Supplementary Figures [file 41598_2019_49281_MOESM1_ESM.docx]

**Improving seasonal forecasts of air temperature using a genetic algorithm**

J. V. Ratnam^1*^, H.A. Dijkstra^2^, Takeshi Doi^1^, Yushi Morioka^1^, Masami Nonaka^1^, Swadhin K. Behera^1^

^1^Application Laboratory, Japan Agency for Marine-Earth Science and Technology, Yokohama Japan

^2^Institute for Marine and Atmospheric research, Utrecht university, Utrecht, The Netherlands

**Supplementary information**

The supplementary figures referred in the main article are presented in this document.

**List of Supplementary figures**

Fig S1: Spatial distribution of ACC values between the 24 members of SINTEX-F2 and CRU air temperature anomalies for the month of June.

The figure was prepared using The NCAR Command Language (version 6.4.0) [Software]. (2017). Boulder, Colorado: UCAR/NCAR/CISL/TDD. <http://dx.doi.org/10.5065/D6WD3XH5>

Fig S2: Spatial distribution of ACC values between the 24 members of SINTEX-F2 and CRU air temperature anomalies for the month of July.

The figure was prepared using The NCAR Command Language (version 6.4.0) [Software]. (2017). Boulder, Colorado: UCAR/NCAR/CISL/TDD. <http://dx.doi.org/10.5065/D6WD3XH5>

Fig S3: Spatial distribution of ACC values between the 24 members of SINTEX-F2 and CRU air temperature anomalies for the month of August.

The figure was prepared using The NCAR Command Language (version 6.4.0) [Software]. (2017). Boulder, Colorado: UCAR/NCAR/CISL/TDD. <http://dx.doi.org/10.5065/D6WD3XH5>

Fig S4: a) Spatial distribution of ACC values between OIV2 SST anomalies and anomalies of SINTEX-F2 forecast for the month of June. b) and c) same as a) but for the months of July and August respectively.

The figure was prepared using The NCAR Command Language (version 6.4.0) [Software]. (2017). Boulder, Colorado: UCAR/NCAR/CISL/TDD. <http://dx.doi.org/10.5065/D6WD3XH5>

Fig S5: a) Spatial distribution of ACC values between ERA-Interim estimated 200hPa velocity potential anomalies and anomalies of SINTEX-F2 forecast for the month of June. b) and c) same as a) but for the months of July and August respectively.

The figure was prepared using The NCAR Command Language (version 6.4.0) [Software]. (2017). Boulder, Colorado: UCAR/NCAR/CISL/TDD. <http://dx.doi.org/10.5065/D6WD3XH5>

Fig S6: Spatial distribution of ACC values between the 24 members of SINTEX-F2 and ERA-Interim 200hPa streamfunction anomalies for the month of June.

The figure was prepared using The NCAR Command Language (version 6.4.0) [Software]. (2017). Boulder, Colorado: UCAR/NCAR/CISL/TDD. <http://dx.doi.org/10.5065/D6WD3XH5>

Fig S7: Spatial distribution of weights generated by the genetic algorithm for the 24-members of SINTEX-F2 for the month of June.

The figure was prepared using The NCAR Command Language (version 6.4.0) [Software]. (2017). Boulder, Colorado: UCAR/NCAR/CISL/TDD. <http://dx.doi.org/10.5065/D6WD3XH5>

Fig S8.a) Spatial distribution of ACC values of SINTEX-F2 2m-air temperature anomalies with respect to CRU anomalies for the month of June using the 3-fold cross validation technique. b) same as a) but for SINTEX-F2ga c) Difference in ACC values between SINTEX-F2ga and SINTEX-F2. The regions of significant (at 90% using Student’s 2-tailed t-test) ACC values are hashed. The rectangular boxes in c) are the regions of significant differences in ACC values between SINTEX-F2ga and SINTEX-F2.

The figure was prepared using The NCAR Command Language (version 6.4.0) [Software]. (2017). Boulder, Colorado: UCAR/NCAR/CISL/TDD. <http://dx.doi.org/10.5065/D6WD3XH5>

Fig S9: a), d), g) and j) spatial distribution of CRU air-temperature (^o^C) anomalies over Australia in June 1989, 1996, 2007 and 2009 respectively. b), e), h) and k) spatial distribution of SINTEX-F2 2m-air temperature (^o^C) anomalies over Australia in June 1989, 1996, 2007 and 2009 respectively. c), f), i) and l) spatial distribution of SINTEX-F2ga 2m-air temperature (^o^C) anomalies over Australia in June 1989, 1996, 2007 and 2009 respectively. The region of analysis is shows as rectangular box in the panels.

The figure was prepared using The NCAR Command Language (version 6.4.0) [Software]. (2017). Boulder, Colorado: UCAR/NCAR/CISL/TDD. <http://dx.doi.org/10.5065/D6WD3XH5>

Fig S10: Spatial distribution of ACC values between the 24 members of SINTEX-F2 and ERA-Interim 200hPa streamfunction anomalies for the month of July.

The figure was prepared using The NCAR Command Language (version 6.4.0) [Software]. (2017). Boulder, Colorado: UCAR/NCAR/CISL/TDD. <http://dx.doi.org/10.5065/D6WD3XH5>

Fig S11: Spatial distribution of weights generated by the genetic algorithm for the 24-members of SINTEX-F2 for the month of July.

The figure was prepared using The NCAR Command Language (version 6.4.0) [Software]. (2017). Boulder, Colorado: UCAR/NCAR/CISL/TDD. <http://dx.doi.org/10.5065/D6WD3XH5>

Fig S12.a) Spatial distribution of ACC values of SINTEX-F2 2m-air temperature anomalies with respect to CRU anomalies for the month of July using the 3-fold cross validation technique. b) same as a) but for SINTEX-F2ga c) Difference in ACC values between SINTEX-F2ga and SINTEX-F2. The regions of significant (at 90% using Student’s 2-tailed t-test) ACC values are hashed. The rectangular boxes in c) are the regions of significant differences in ACC values between SINTEX-F2ga and SINTEX-F2.

The figure was prepared using The NCAR Command Language (version 6.4.0) [Software]. (2017). Boulder, Colorado: UCAR/NCAR/CISL/TDD. <http://dx.doi.org/10.5065/D6WD3XH5>

Fig S13: a) and d) spatial distribution of CRU air-temperature (^o^C) anomalies over North America in July 2011 and 2012 respectively. b), and e) spatial distribution of SINTEX-F2 2m-air temperature (^o^C) anomalies over North America in July 2011 and 2012 respectively. c) and f) spatial distribution of SINTEX-F2ga 2m-air temperature (^o^C) anomalies over North America in July 2011 and 2012 respectively. The region of analysis is shows as rectangular box in the panels.

The figure was prepared using The NCAR Command Language (version 6.4.0) [Software]. (2017). Boulder, Colorado: UCAR/NCAR/CISL/TDD. <http://dx.doi.org/10.5065/D6WD3XH5>

Fig S14: a) and d) spatial distribution of CRU air-temperature (^o^C) anomalies over South America in July 1997 and 2005 respectively. b), and e) spatial distribution of SINTEX-F2 2m-air temperature (^o^C) anomalies over South America in July 1997 and 2005 respectively. c) and f) spatial distribution of SINTEX-F2ga 2m-air temperature (^o^C) anomalies over South America in July 1997 and 2005 respectively. The region of analysis is shows as rectangular box in the panels.

The figure was prepared using The NCAR Command Language (version 6.4.0) [Software]. (2017). Boulder, Colorado: UCAR/NCAR/CISL/TDD. <http://dx.doi.org/10.5065/D6WD3XH5>

Fig S15: a), d), g), and j) spatial distribution of CRU air-temperature (^o^C) anomalies over South America in July 1995, 2003, 2004 and 2006 respectively. b), e), h) and k) spatial distribution of SINTEX-F2 2m-air temperature (^o^C) anomalies over South America in July 1995, 2003, 2004 and 2006 respectively. c), f), i) and l) spatial distribution of SINTEX-F2ga 2m-air temperature (^o^C) anomalies over South America in July 1995, 2003, 2004 and 2006 respectively. The region of analysis is shows as rectangular box in the panels.

The figure was prepared using The NCAR Command Language (version 6.4.0) [Software]. (2017). Boulder, Colorado: UCAR/NCAR/CISL/TDD. <http://dx.doi.org/10.5065/D6WD3XH5>

Fig S16: Spatial distribution of ACC values between the 24 members of SINTEX-F2 and ERA-Interim 200hPa streamfunction anomalies for the month of August.

The figure was prepared using The NCAR Command Language (version 6.4.0) [Software]. (2017). Boulder, Colorado: UCAR/NCAR/CISL/TDD. <http://dx.doi.org/10.5065/D6WD3XH5>

Fig S17: Spatial distribution of weights generated by the genetic algorithm for the 24-members of SINTEX-F2 for the month of August.

The figure was prepared using The NCAR Command Language (version 6.4.0) [Software]. (2017). Boulder, Colorado: UCAR/NCAR/CISL/TDD. <http://dx.doi.org/10.5065/D6WD3XH5>

Fig S18.a) Spatial distribution of ACC values of SINTEX-F2 2m-air temperature anomalies with respect to CRU anomalies for the month of August using the 3-fold cross validation technique. b) same as a) but for SINTEX-F2ga c) Difference in ACC values between SINTEX-F2ga and SINTEX-F2. The regions of significant (at 90% using Student’s 2-tailed t-test) ACC values are hashed. The rectangular boxes in c) are the regions of significant differences in ACC values between SINTEX-F2ga and SINTEX-F2.

The figure was prepared using The NCAR Command Language (version 6.4.0) [Software]. (2017). Boulder, Colorado: UCAR/NCAR/CISL/TDD. <http://dx.doi.org/10.5065/D6WD3XH5>

Fig S19: a) spatial distribution of CRU air-temperature (^o^C) anomalies over North America in Aug 2010. b) spatial distribution of SINTEX-F2 2m-air temperature (^o^C) anomalies over North America in August 2010. c) spatial distribution of SINTEX-F2ga 2m-air temperature (^o^C) anomalies over North America in August 2010. The region of analysis is shows as rectangular box in the panels.

The figure was prepared using The NCAR Command Language (version 6.4.0) [Software]. (2017). Boulder, Colorado: UCAR/NCAR/CISL/TDD. <http://dx.doi.org/10.5065/D6WD3XH5>

Fig S20.a) Spatial distribution of ACC values of SINTEX-F2 2m-air temperature anomalies with respect to CRU anomalies for the month of Dec. b) same as a) but for SINTEX-F2ga c) Difference in ACC values between SINTEX-F2ga and SINTEX-F2. The regions of significant (at 90% using Student’s 2-tailed t-test) ACC values are hashed. The rectangular boxes in c) are the regions of significant differences in ACC values between SINTEX-F2ga and SINTEX-F2.

The figure was prepared using The NCAR Command Language (version 6.4.0) [Software]. (2017). Boulder, Colorado: UCAR/NCAR/CISL/TDD. <http://dx.doi.org/10.5065/D6WD3XH5>

Fig S21. Same as Fig S20 but for Jan.

The figure was prepared using The NCAR Command Language (version 6.4.0) [Software]. (2017). Boulder, Colorado: UCAR/NCAR/CISL/TDD. <http://dx.doi.org/10.5065/D6WD3XH5>

Fig S22. Same as Fig S20 but for Feb.

The figure was prepared using The NCAR Command Language (version 6.4.0) [Software]. (2017). Boulder, Colorado: UCAR/NCAR/CISL/TDD. <http://dx.doi.org/10.5065/D6WD3XH5>


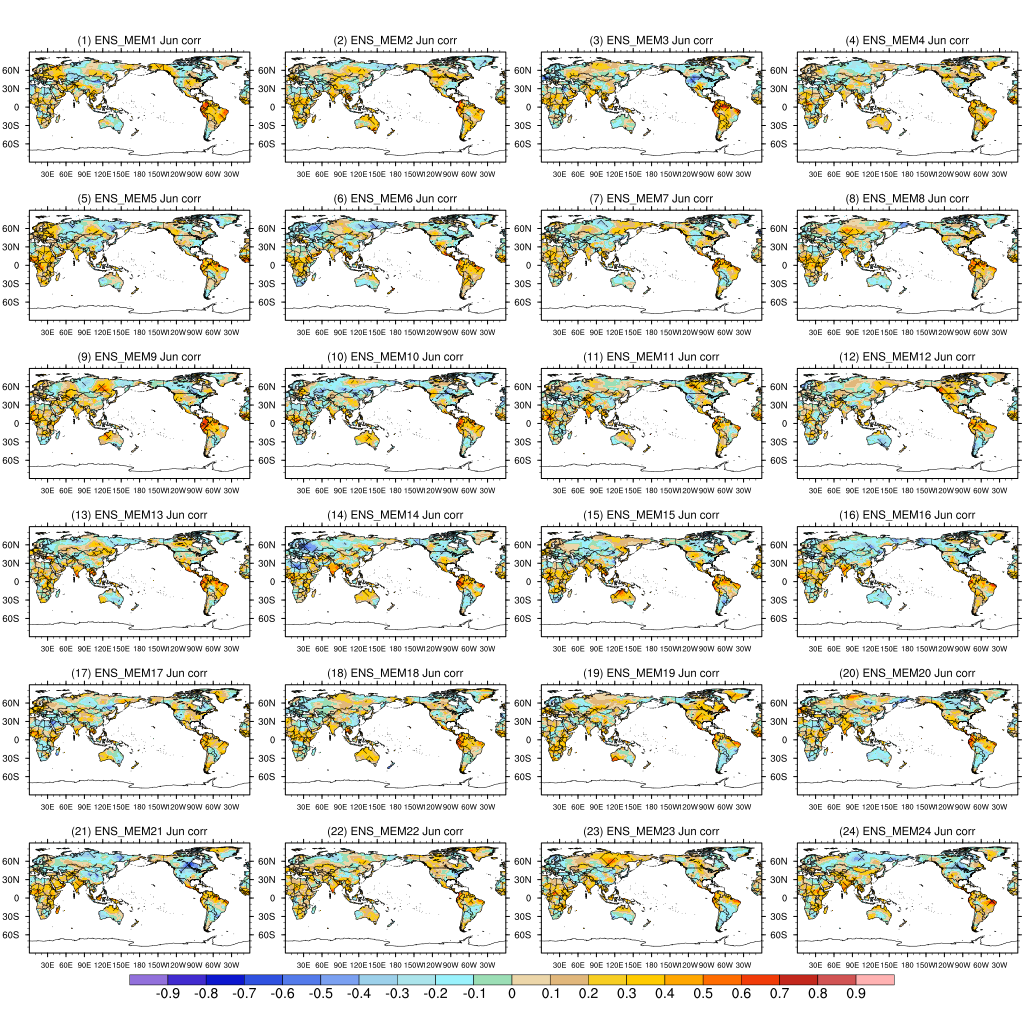


Fig S1: Spatial distribution of ACC values between the 24 members of SINTEX-F2 and CRU air temperature anomalies for the month of June.

The figure was prepared using The NCAR Command Language (version 6.4.0) [Software]. (2017). Boulder, Colorado: UCAR/NCAR/CISL/TDD. <http://dx.doi.org/10.5065/D6WD3XH5>


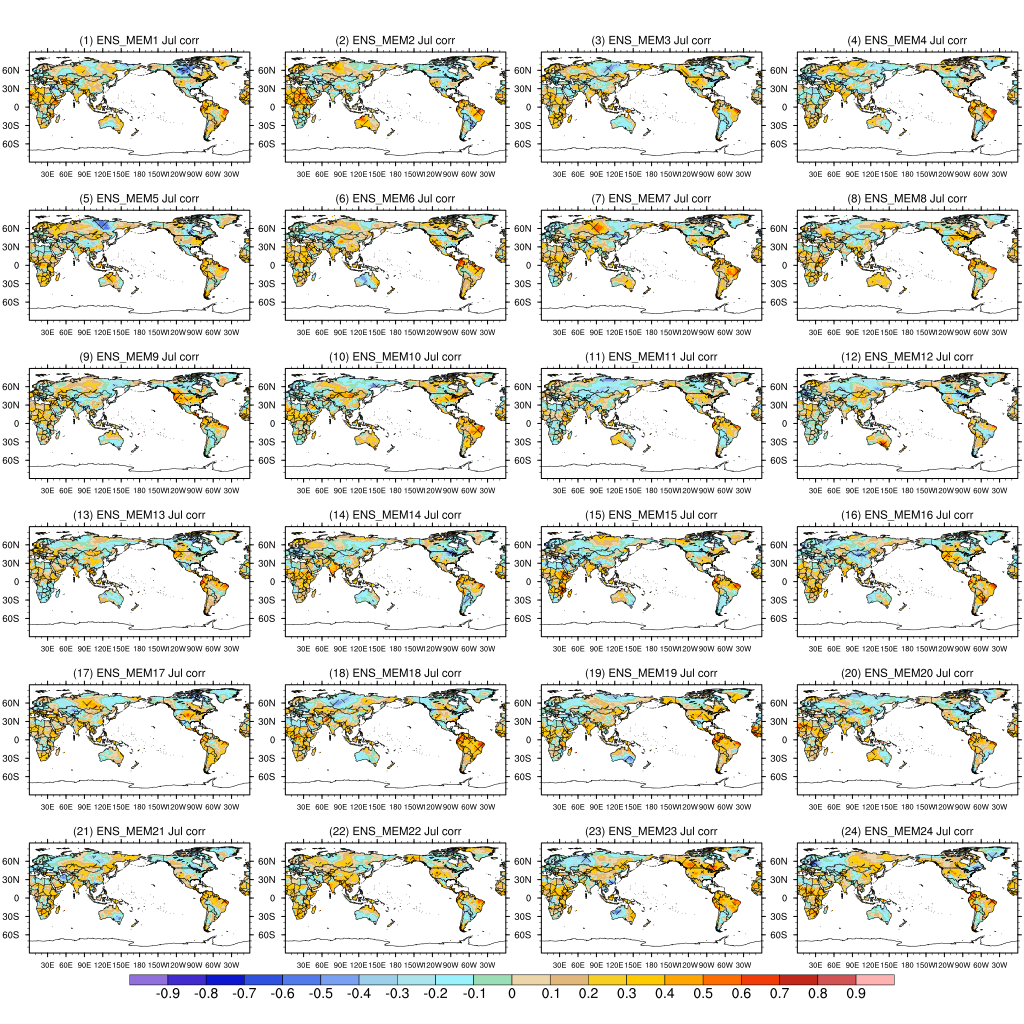


Fig S2: Spatial distribution of ACC values between the 24 members of SINTEX-F2 and CRU air temperature anomalies for the month of July.

The figure was prepared using The NCAR Command Language (version 6.4.0) [Software]. (2017). Boulder, Colorado: UCAR/NCAR/CISL/TDD. <http://dx.doi.org/10.5065/D6WD3XH5>


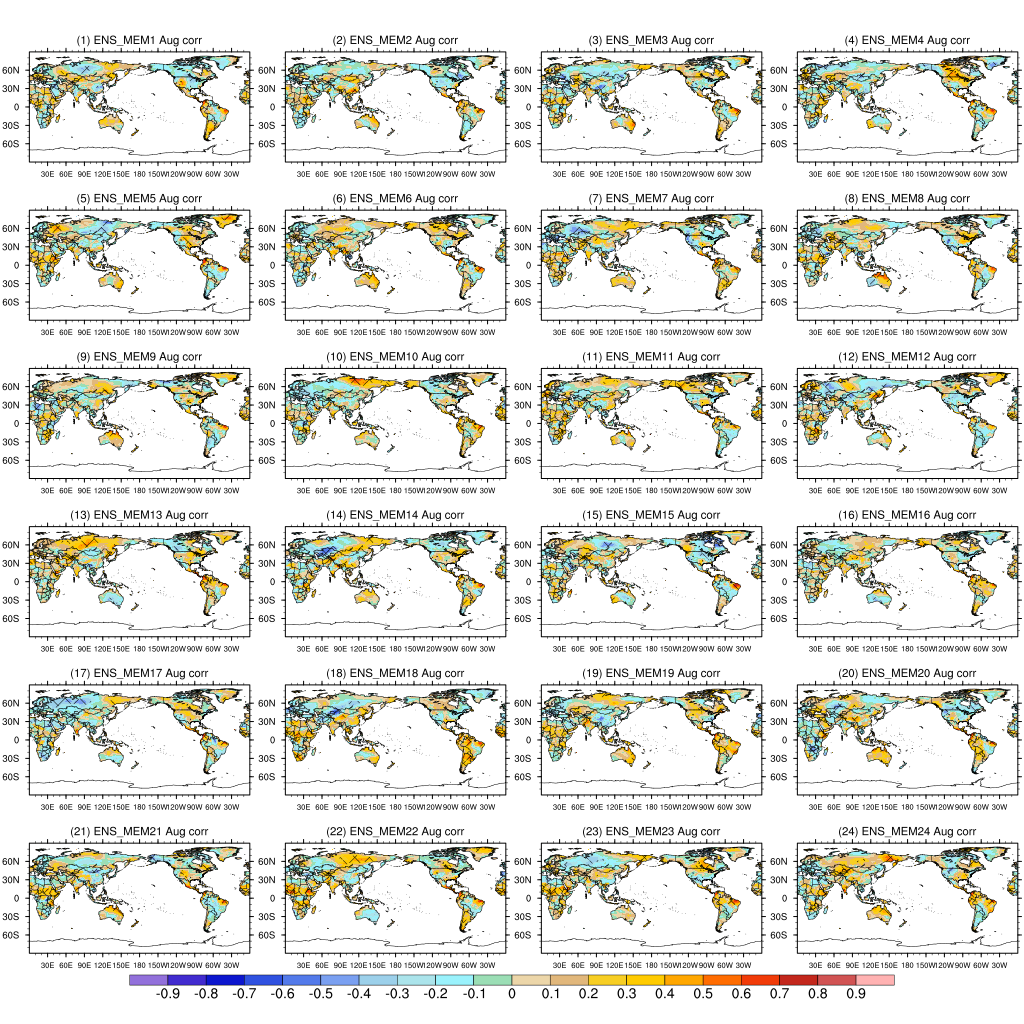


Fig S3: Spatial distribution of ACC values between the 24 members of SINTEX-F2 and CRU air temperature anomalies for the month of August.

The figure was prepared using The NCAR Command Language (version 6.4.0) [Software]. (2017). Boulder, Colorado: UCAR/NCAR/CISL/TDD. <http://dx.doi.org/10.5065/D6WD3XH5>


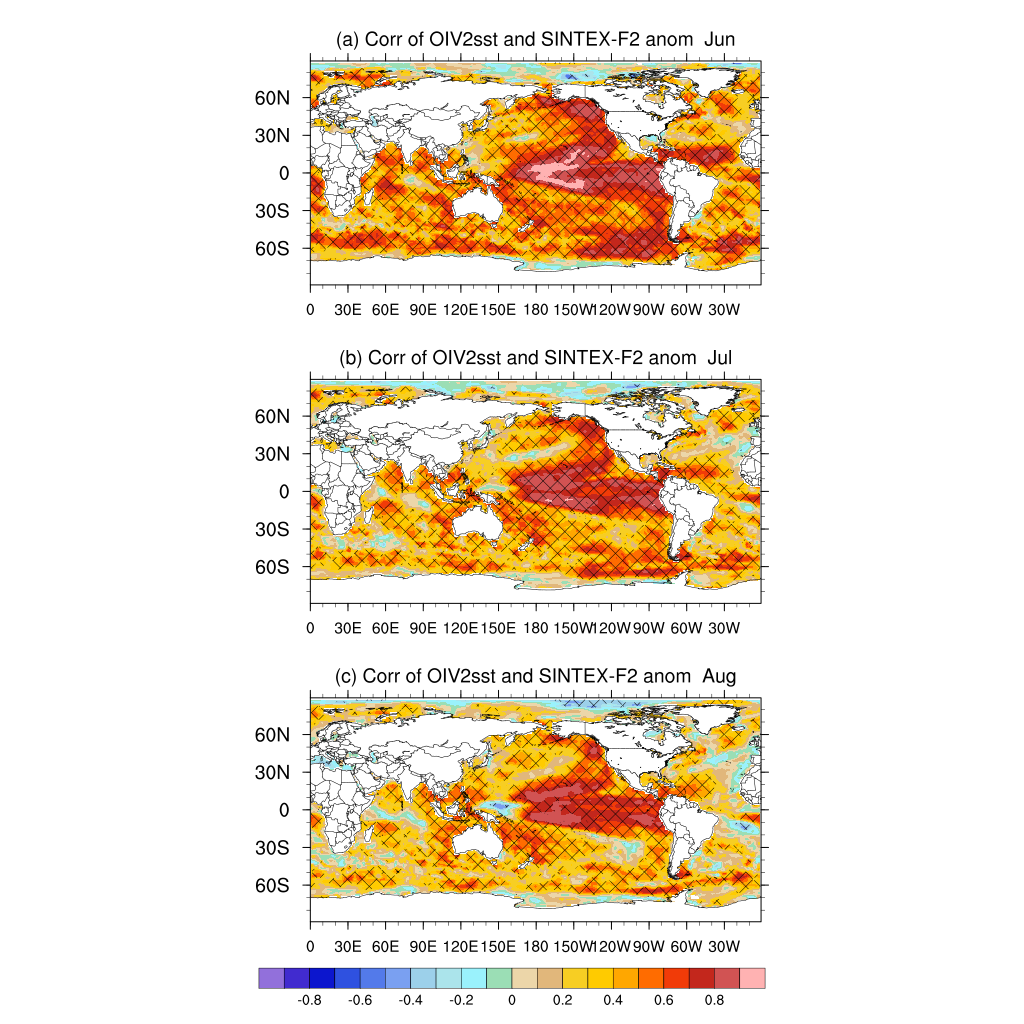


Fig S4: a) Spatial distribution of ACC values between OIV2 SST anomalies and anomalies of SINTEX-F2 forecast for the month of June. b) and c) same as a) but for the months of July and August respectively.

The figure was prepared using The NCAR Command Language (version 6.4.0) [Software]. (2017). Boulder, Colorado: UCAR/NCAR/CISL/TDD. <http://dx.doi.org/10.5065/D6WD3XH5>


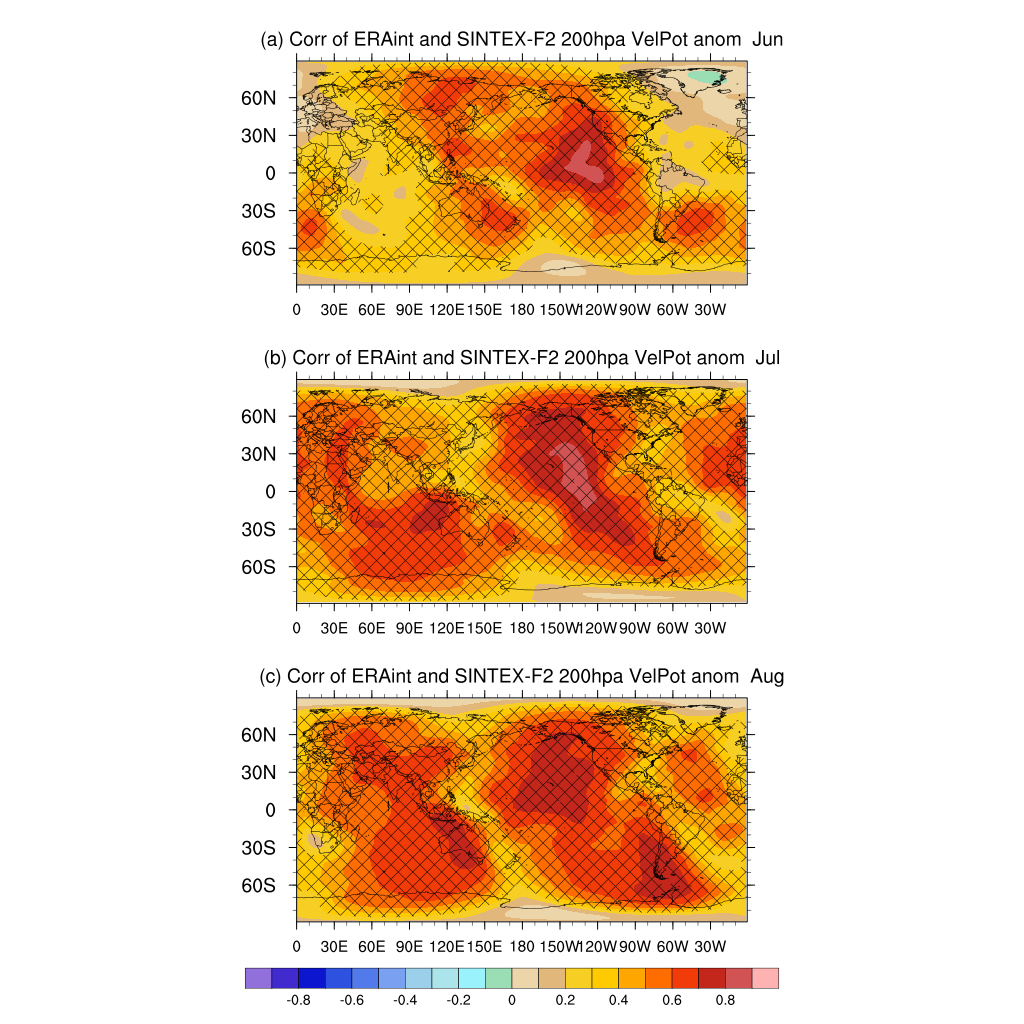


Fig S5: a) Spatial distribution of ACC values between ERA-Interim estimated 200hPa velocity potential anomalies and anomalies of SINTEX-F2 forecast for the month of June. b) and c) same as a) but for the months of July and August respectively.

The figure was prepared using The NCAR Command Language (version 6.4.0) [Software]. (2017). Boulder, Colorado: UCAR/NCAR/CISL/TDD. <http://dx.doi.org/10.5065/D6WD3XH5>


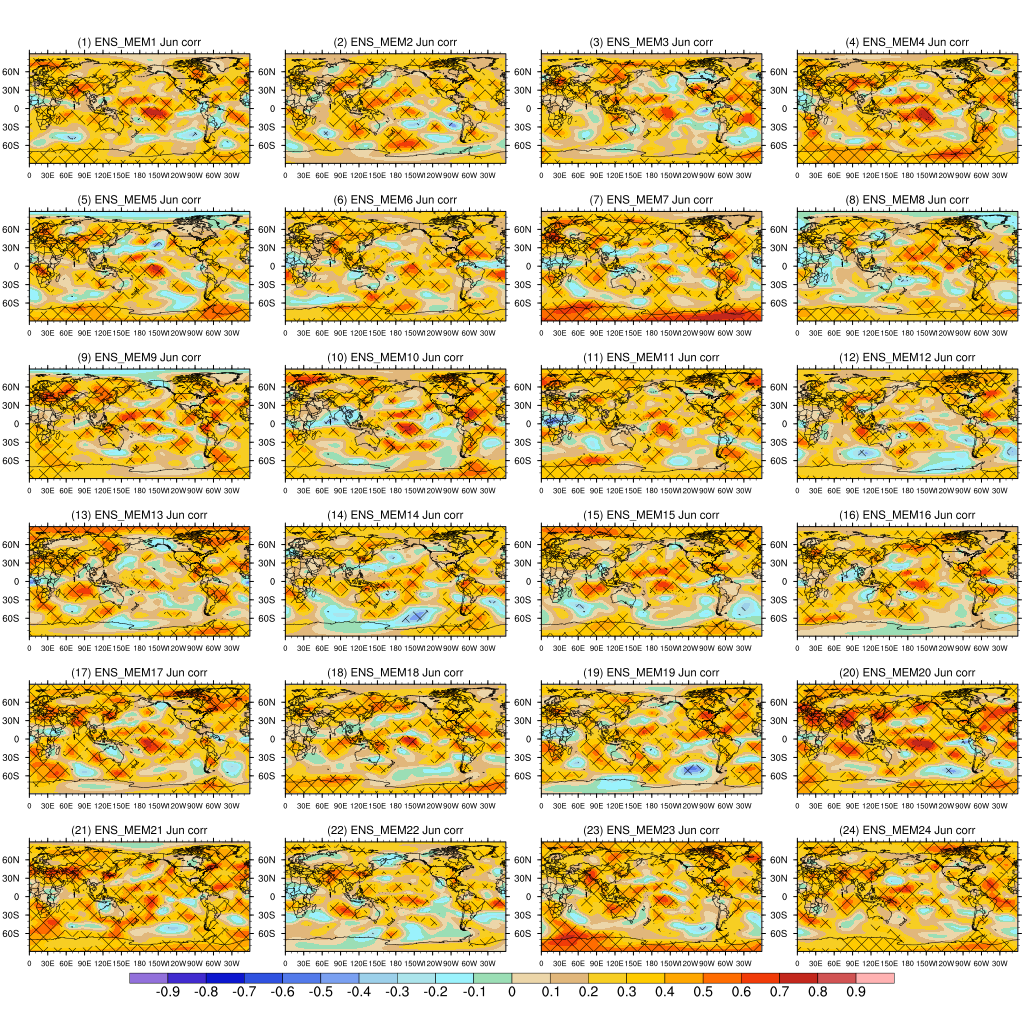


Fig S6: Spatial distribution of ACC values between the 24 members of SINTEX-F2 and ERA-Interim 200hPa streamfunction anomalies for the month of June.

The figure was prepared using The NCAR Command Language (version 6.4.0) [Software]. (2017). Boulder, Colorado: UCAR/NCAR/CISL/TDD. <http://dx.doi.org/10.5065/D6WD3XH5>


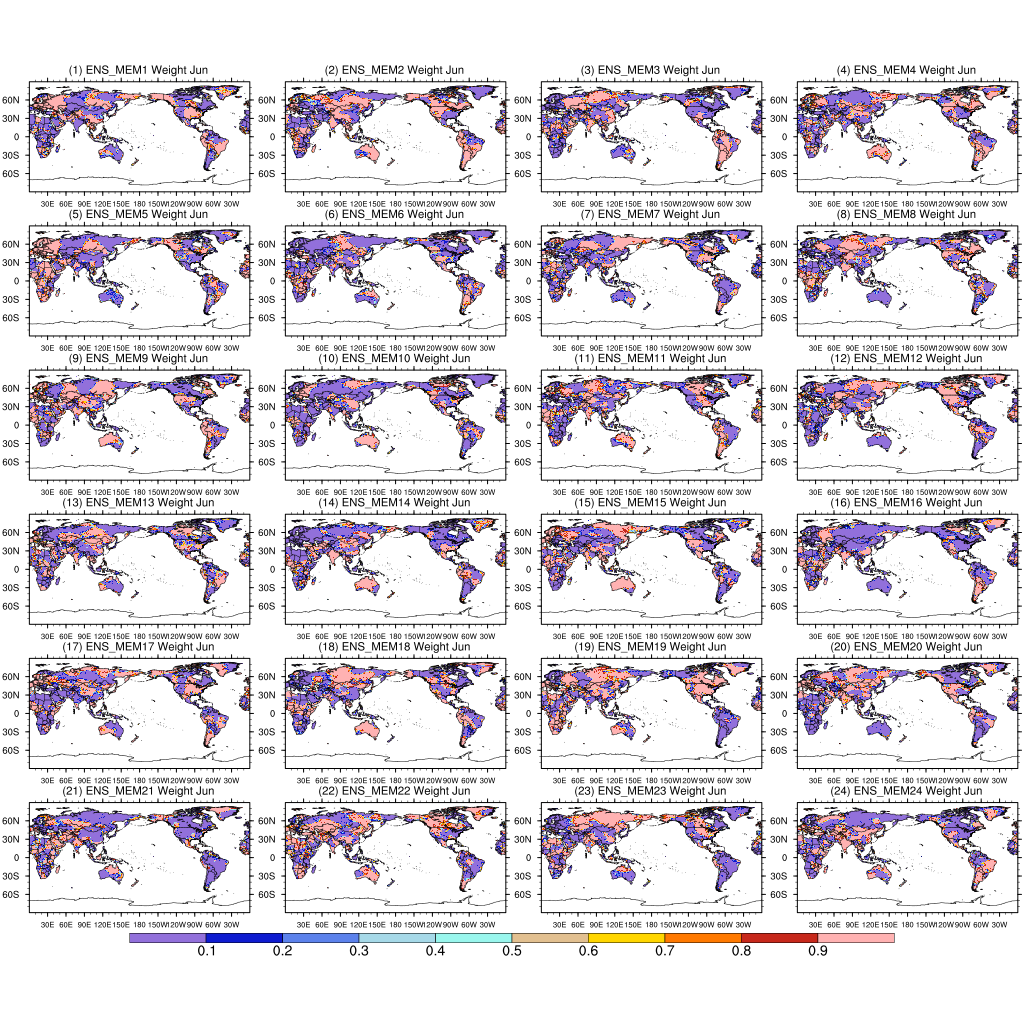


Fig S7: Spatial distribution of weights generated by the genetic algorithm for the 24-members of SINTEX-F2 for the month of June.

The figure was prepared using The NCAR Command Language (version 6.4.0) [Software]. (2017). Boulder, Colorado: UCAR/NCAR/CISL/TDD. <http://dx.doi.org/10.5065/D6WD3XH5>


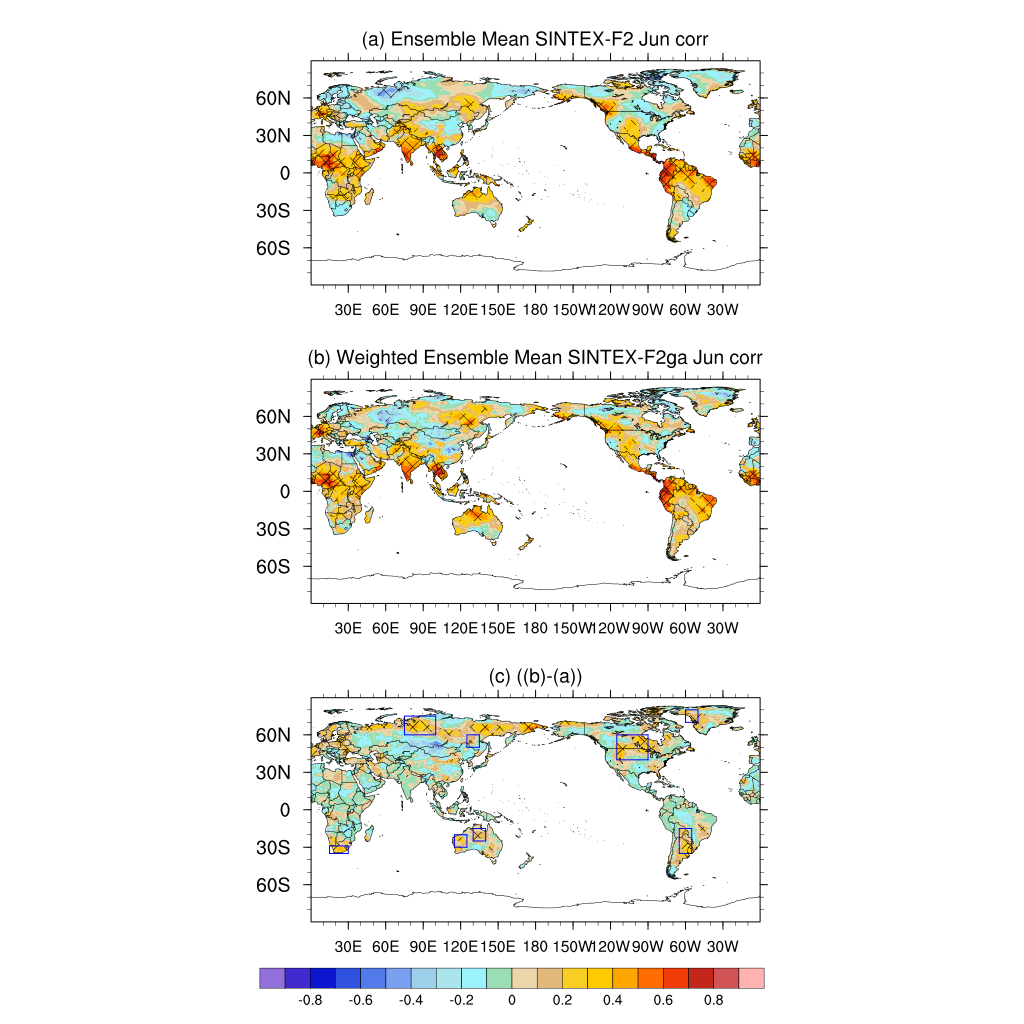


Fig S8.a) Spatial distribution of ACC values of SINTEX-F2 2m-air temperature anomalies with respect to CRU anomalies for the month of June using the 3-fold cross validation technique. b) same as a) but for SINTEX-F2ga c) Difference in ACC values between SINTEX-F2ga and SINTEX-F2. The regions of significant (at 90% using Student’s 2-tailed t-test) ACC values are hashed. The rectangular boxes in c) are the regions of significant differences in ACC values between SINTEX-F2ga and SINTEX-F2.

The figure was prepared using The NCAR Command Language (version 6.4.0) [Software]. (2017). Boulder, Colorado: UCAR/NCAR/CISL/TDD. <http://dx.doi.org/10.5065/D6WD3XH5>


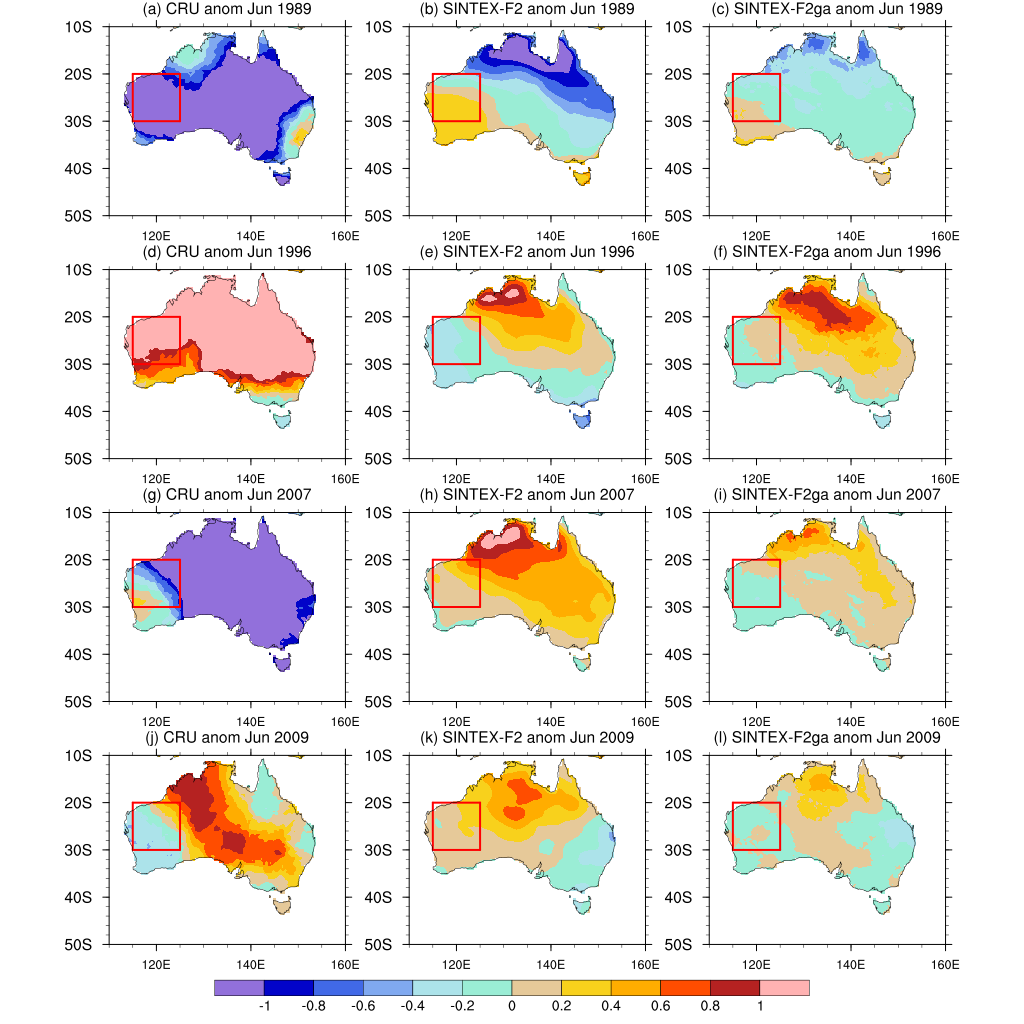


Fig S9: a), d), g) and j) spatial distribution of CRU air-temperature (^o^C) anomalies over Australia in June 1989, 1996, 2007 and 2009 respectively. b), e), h) and k) spatial distribution of SINTEX-F2 2m-air temperature (^o^C) anomalies over Australia in June 1989, 1996, 2007 and 2009 respectively. c), f), i) and l) spatial distribution of SINTEX-F2ga 2m-air temperature (^o^C) anomalies over Australia in June 1989, 1996, 2007 and 2009 respectively. The region of analysis is shows as rectangular box in the panels.

The figure was prepared using The NCAR Command Language (version 6.4.0) [Software]. (2017). Boulder, Colorado: UCAR/NCAR/CISL/TDD. <http://dx.doi.org/10.5065/D6WD3XH5>


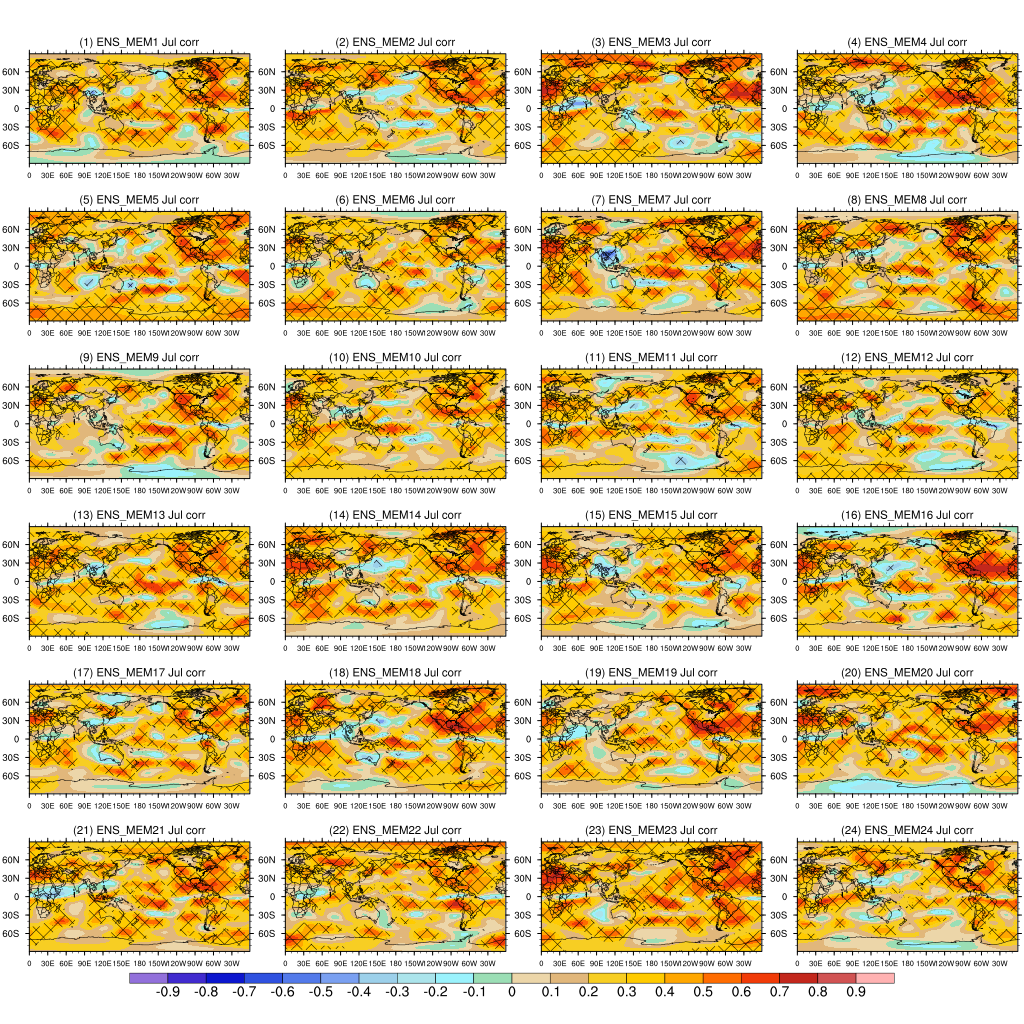


Fig S10: Spatial distribution of ACC values between the 24 members of SINTEX-F2 and ERA-Interim 200hPa streamfunction anomalies for the month of July.

The figure was prepared using The NCAR Command Language (version 6.4.0) [Software]. (2017). Boulder, Colorado: UCAR/NCAR/CISL/TDD. <http://dx.doi.org/10.5065/D6WD3XH5>


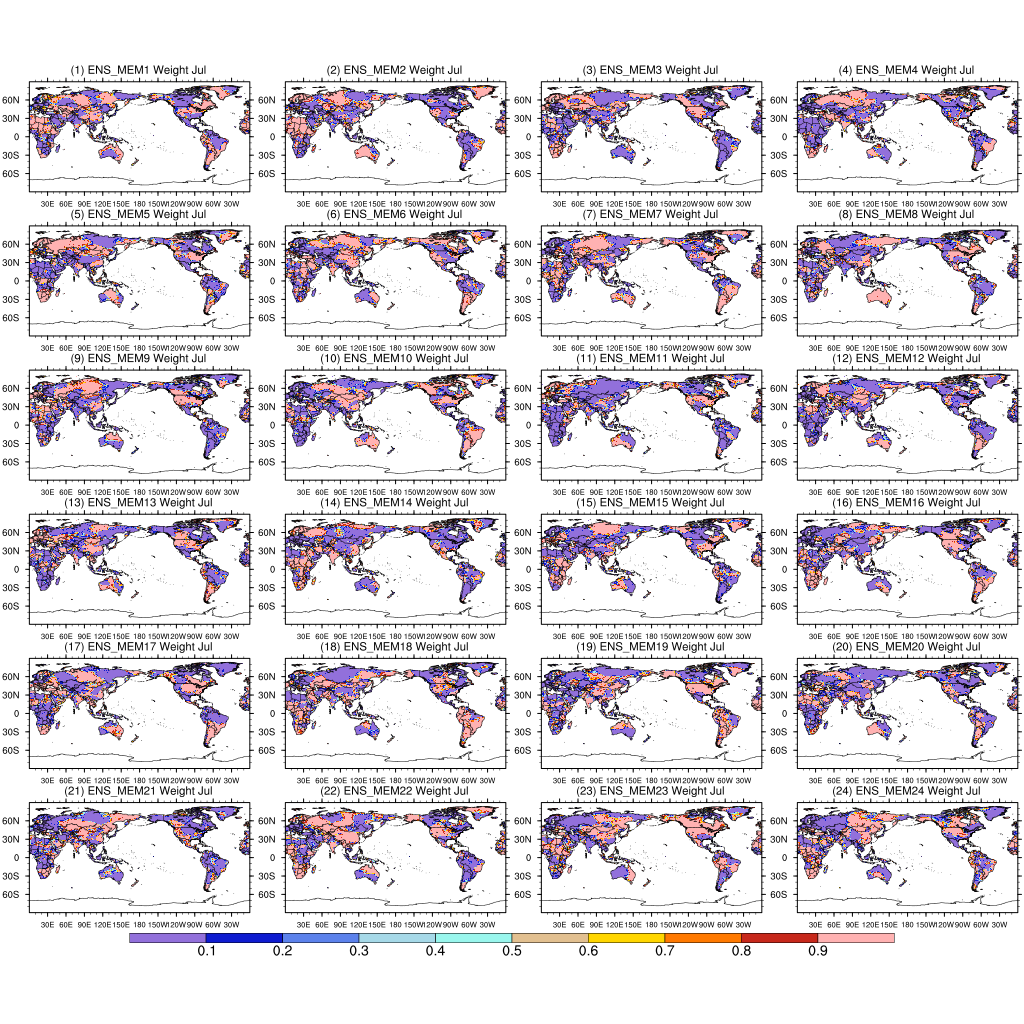


Fig S11: Spatial distribution of weights generated by the genetic algorithm for the 24-members of SINTEX-F2 for the month of July.

The figure was prepared using The NCAR Command Language (version 6.4.0) [Software]. (2017). Boulder, Colorado: UCAR/NCAR/CISL/TDD. <http://dx.doi.org/10.5065/D6WD3XH5>


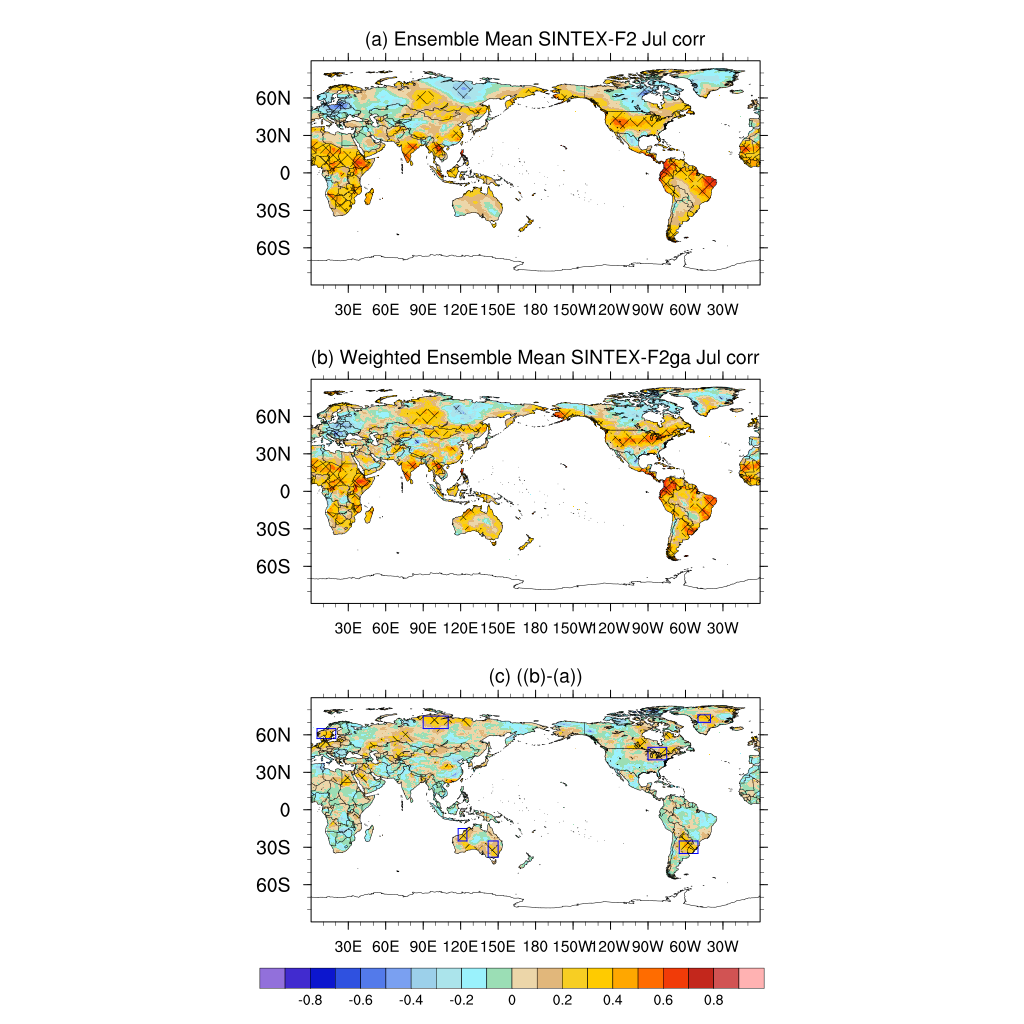


Fig S12.a) Spatial distribution of ACC values of SINTEX-F2 2m-air temperature anomalies with respect to CRU anomalies for the month of July using the 3-fold cross validation technique. b) same as a) but for SINTEX-F2ga c) Difference in ACC values between SINTEX-F2ga and SINTEX-F2. The regions of significant (at 90% using Student’s 2-tailed t-test) ACC values are hashed. The rectangular boxes in c) are the regions of significant differences in ACC values between SINTEX-F2ga and SINTEX-F2.

The figure was prepared using The NCAR Command Language (version 6.4.0) [Software]. (2017). Boulder, Colorado: UCAR/NCAR/CISL/TDD. <http://dx.doi.org/10.5065/D6WD3XH5>


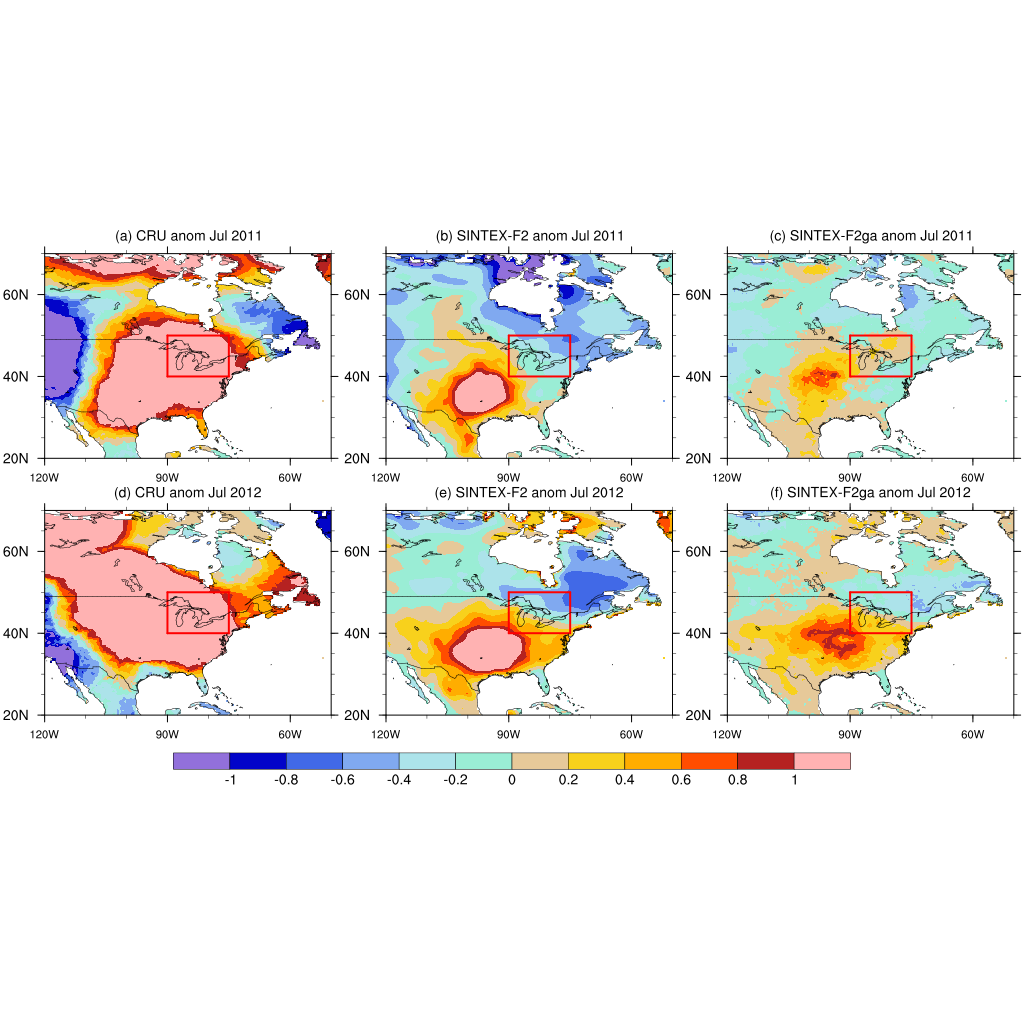


Fig S13: a) and d) spatial distribution of CRU air-temperature (^o^C) anomalies over North America in July 2011 and 2012 respectively. b), and e) spatial distribution of SINTEX-F2 2m-air temperature (^o^C) anomalies over North America in July 2011 and 2012 respectively. c) and f) spatial distribution of SINTEX-F2ga 2m-air temperature (^o^C) anomalies over North America in July 2011 and 2012 respectively. The region of analysis is shows as rectangular box in the panels.

The figure was prepared using The NCAR Command Language (version 6.4.0) [Software]. (2017). Boulder, Colorado: UCAR/NCAR/CISL/TDD. <http://dx.doi.org/10.5065/D6WD3XH5>


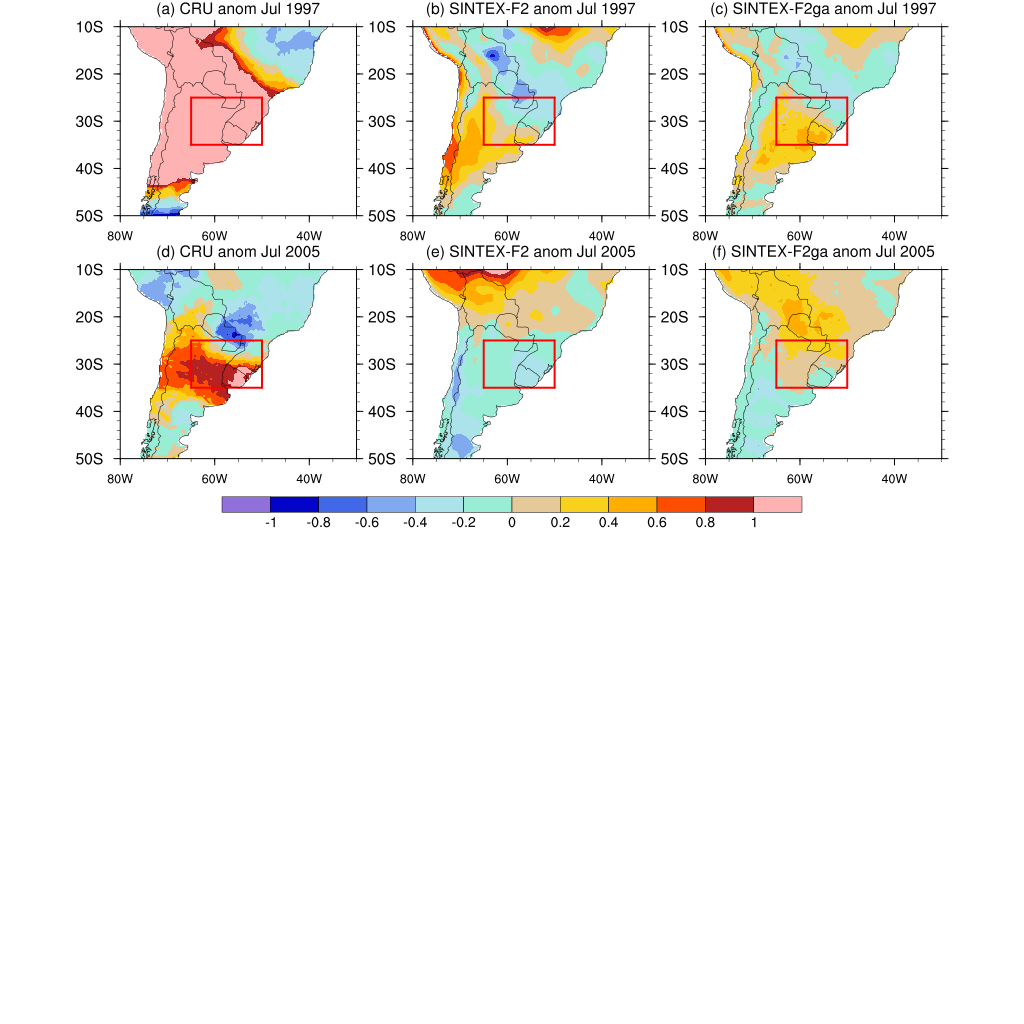


Fig S14: a) and d) spatial distribution of CRU air-temperature (^o^C) anomalies over South America in July 1997 and 2005 respectively. b), and e) spatial distribution of SINTEX-F2 2m-air temperature (^o^C) anomalies over South America in July 1997 and 2005 respectively. c) and f) spatial distribution of SINTEX-F2ga 2m-air temperature (^o^C) anomalies over South America in July 1997 and 2005 respectively. The region of analysis is shows as rectangular box in the panels.

The figure was prepared using The NCAR Command Language (version 6.4.0) [Software]. (2017). Boulder, Colorado: UCAR/NCAR/CISL/TDD. <http://dx.doi.org/10.5065/D6WD3XH5>


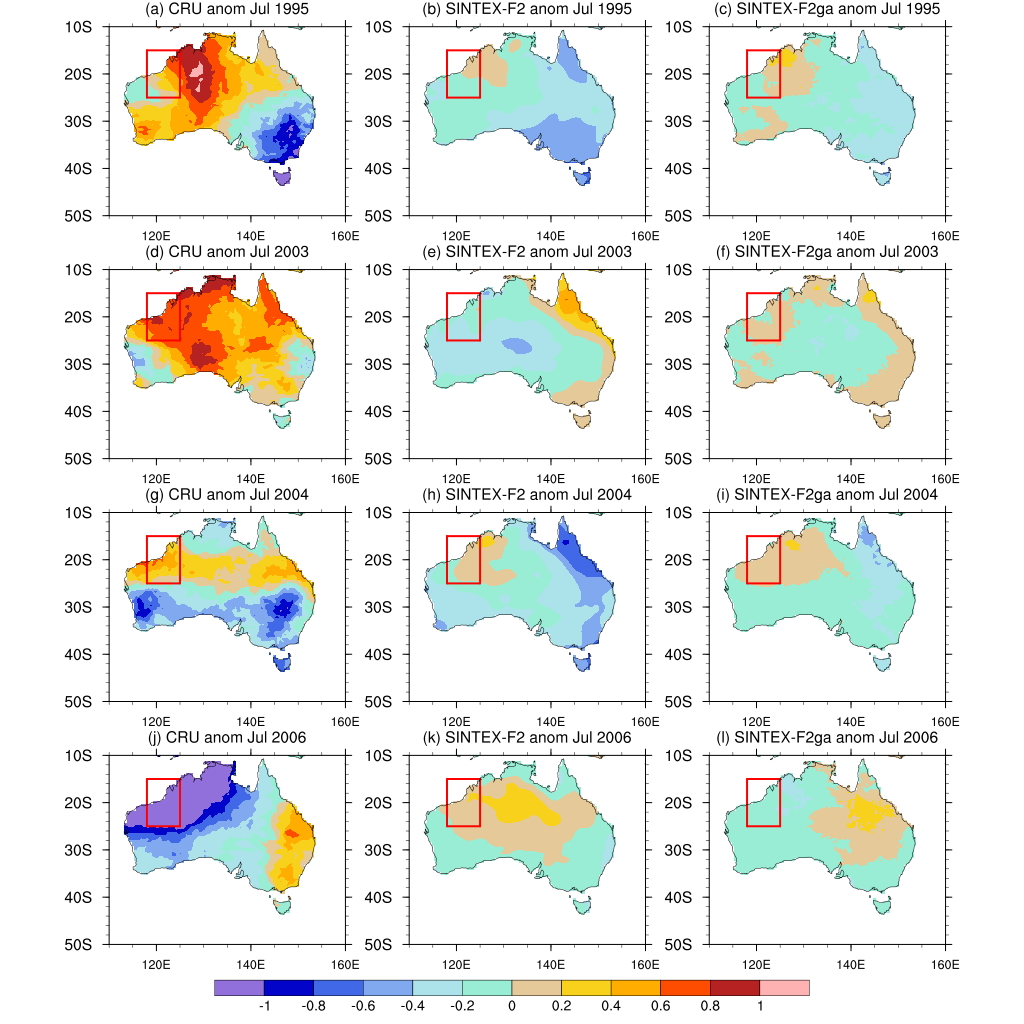


Fig S15: a), d), g), and j) spatial distribution of CRU air-temperature (^o^C) anomalies over South America in July 1995, 2003, 2004 and 2006 respectively. b), e), h) and k) spatial distribution of SINTEX-F2 2m-air temperature (^o^C) anomalies over South America in July 1995, 2003, 2004 and 2006 respectively. c), f), i) and l) spatial distribution of SINTEX-F2ga 2m-air temperature (^o^C) anomalies over South America in July 1995, 2003, 2004 and 2006 respectively. The region of analysis is shows as rectangular box in the panels.

The figure was prepared using The NCAR Command Language (version 6.4.0) [Software]. (2017). Boulder, Colorado: UCAR/NCAR/CISL/TDD. <http://dx.doi.org/10.5065/D6WD3XH5>


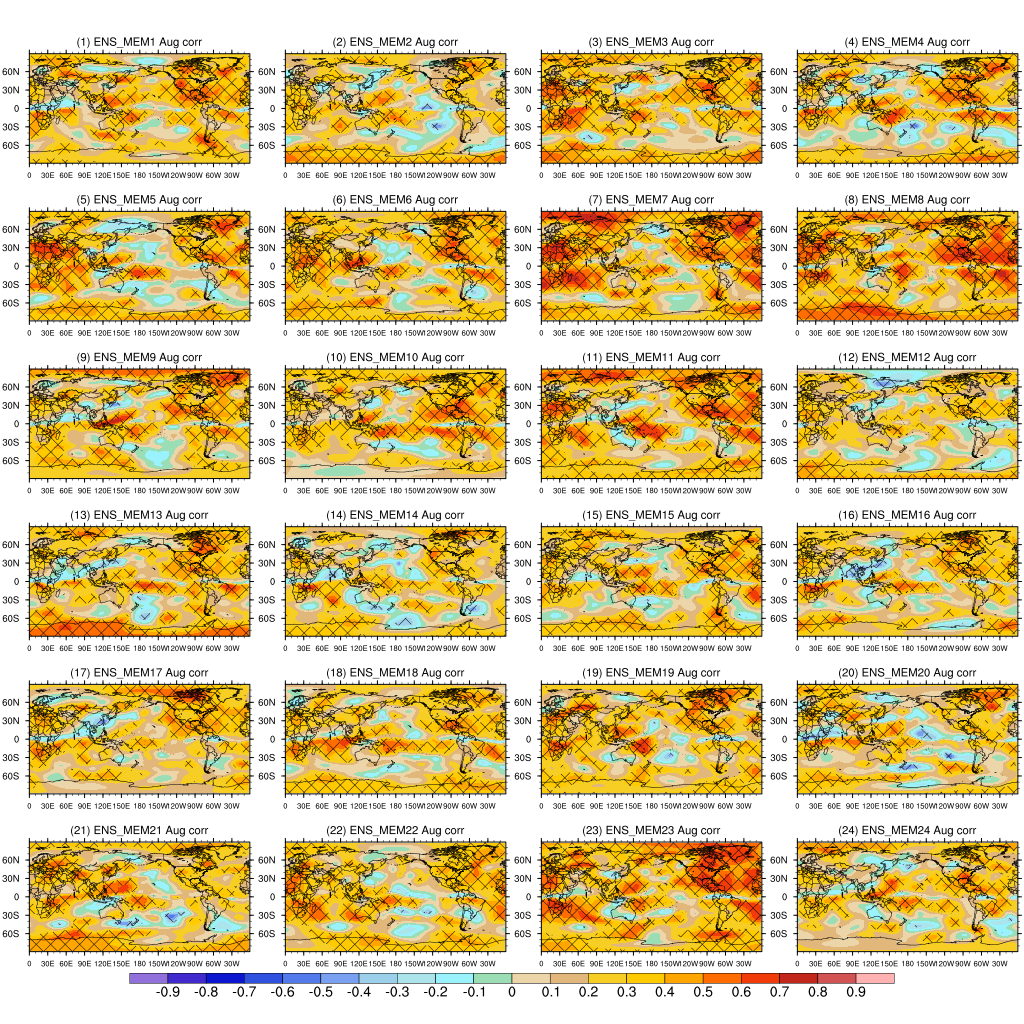


Fig S16: Spatial distribution of ACC values between the 24 members of SINTEX-F2 and ERA-Interim 200hPa streamfunction anomalies for the month of August.

The figure was prepared using The NCAR Command Language (version 6.4.0) [Software]. (2017). Boulder, Colorado: UCAR/NCAR/CISL/TDD. <http://dx.doi.org/10.5065/D6WD3XH5>


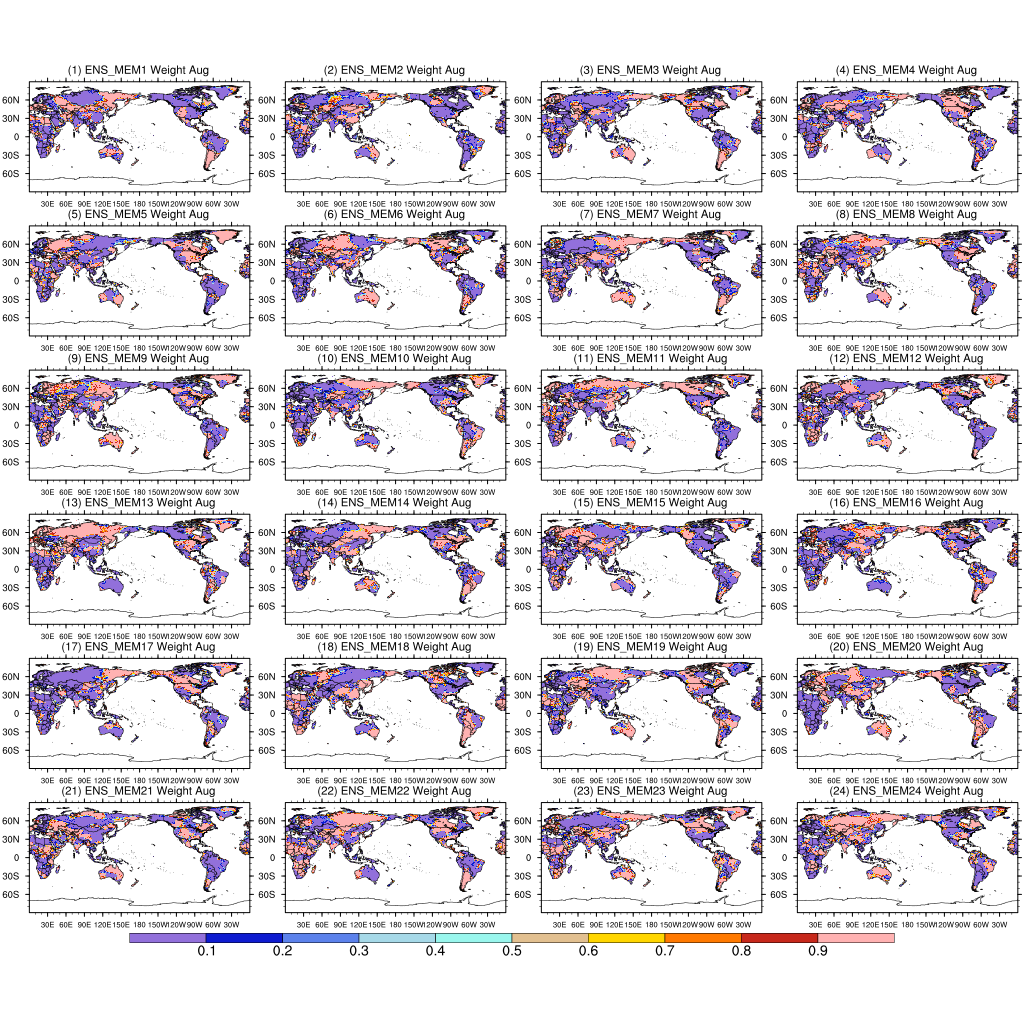


Fig S17: Spatial distribution of weights generated by the genetic algorithm for the 24-members of SINTEX-F2 for the month of August.

The figure was prepared using The NCAR Command Language (version 6.4.0) [Software]. (2017). Boulder, Colorado: UCAR/NCAR/CISL/TDD. <http://dx.doi.org/10.5065/D6WD3XH5>


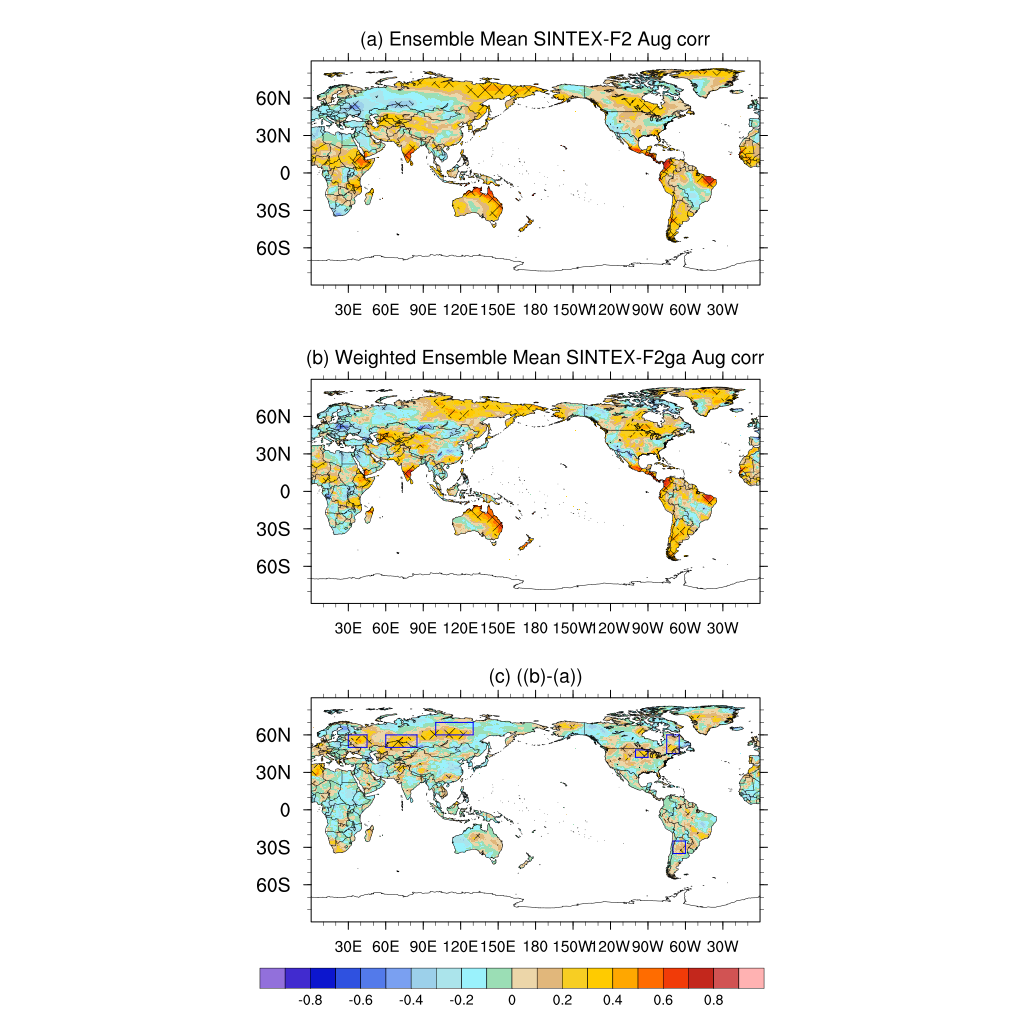


Fig S18.a) Spatial distribution of ACC values of SINTEX-F2 2m-air temperature anomalies with respect to CRU anomalies for the month of August using the 3-fold cross validation technique. b) same as a) but for SINTEX-F2ga c) Difference in ACC values between SINTEX-F2ga and SINTEX-F2. The regions of significant (at 90% using Student’s 2-tailed t-test) ACC values are hashed. The rectangular boxes in c) are the regions of significant differences in ACC values between SINTEX-F2ga and SINTEX-F2.

The figure was prepared using The NCAR Command Language (version 6.4.0) [Software]. (2017). Boulder, Colorado: UCAR/NCAR/CISL/TDD. <http://dx.doi.org/10.5065/D6WD3XH5>


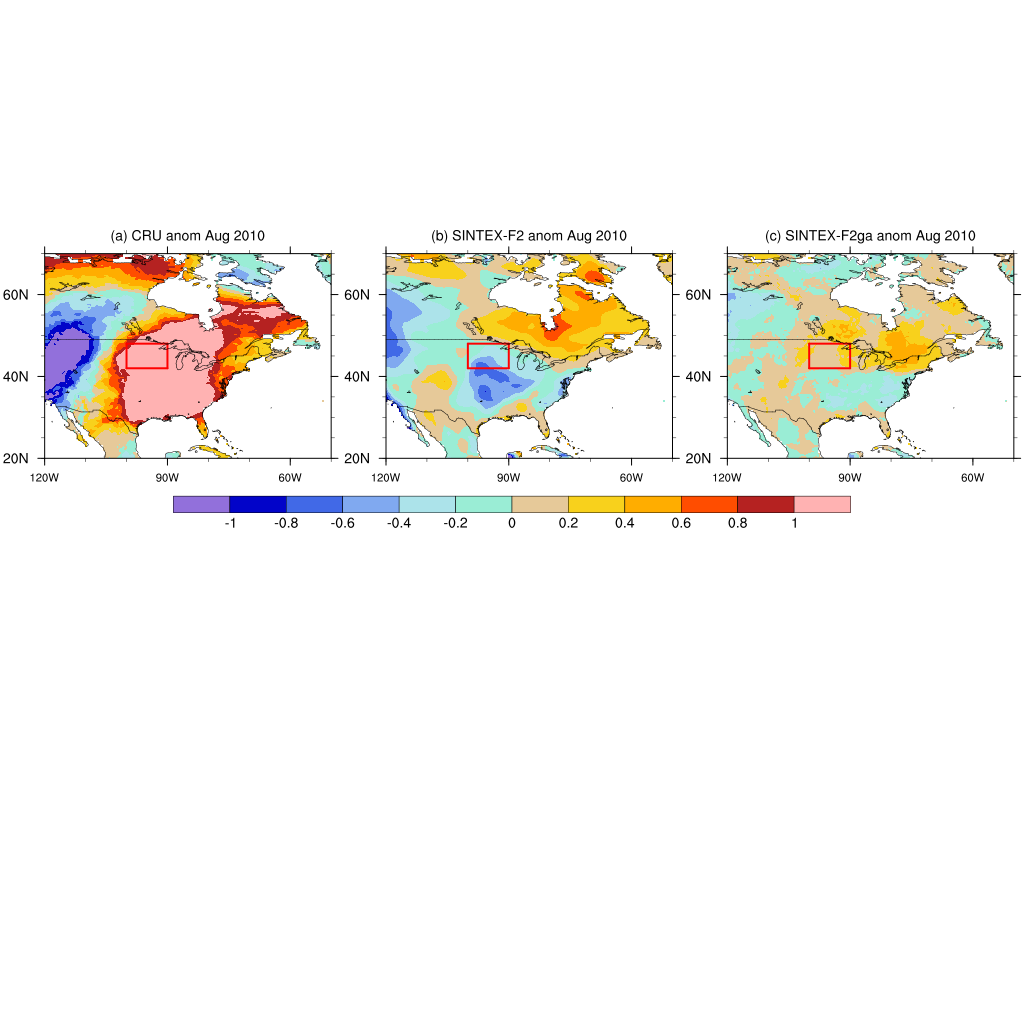


Fig S19: a) spatial distribution of CRU air-temperature (^o^C) anomalies over North America in Aug 2010. b) spatial distribution of SINTEX-F2 2m-air temperature (^o^C) anomalies over North America in August 2010. c) spatial distribution of SINTEX-F2ga 2m-air temperature (^o^C) anomalies over North America in August 2010. The region of analysis is shows as rectangular box in the panels.

The figure was prepared using The NCAR Command Language (version 6.4.0) [Software]. (2017). Boulder, Colorado: UCAR/NCAR/CISL/TDD. <http://dx.doi.org/10.5065/D6WD3XH5>


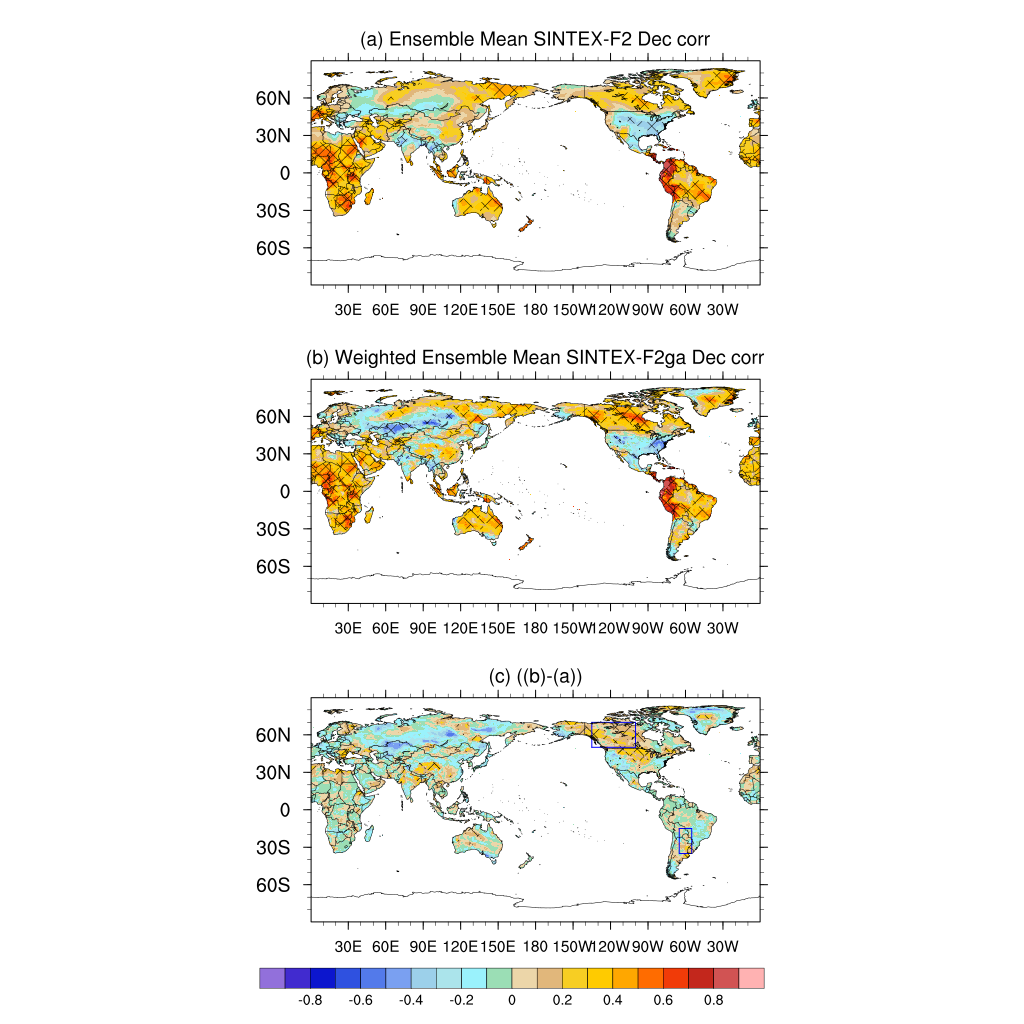


Fig S20.a) Spatial distribution of ACC values of SINTEX-F2 2m-air temperature anomalies with respect to CRU anomalies for the month of Dec. b) same as a) but for SINTEX-F2ga c) Difference in ACC values between SINTEX-F2ga and SINTEX-F2. The regions of significant (at 90% using Student’s 2-tailed t-test) ACC values are hashed. The rectangular boxes in c) are the regions of significant differences in ACC values between SINTEX-F2ga and SINTEX-F2.

The figure was prepared using The NCAR Command Language (version 6.4.0) [Software]. (2017). Boulder, Colorado: UCAR/NCAR/CISL/TDD. <http://dx.doi.org/10.5065/D6WD3XH5>


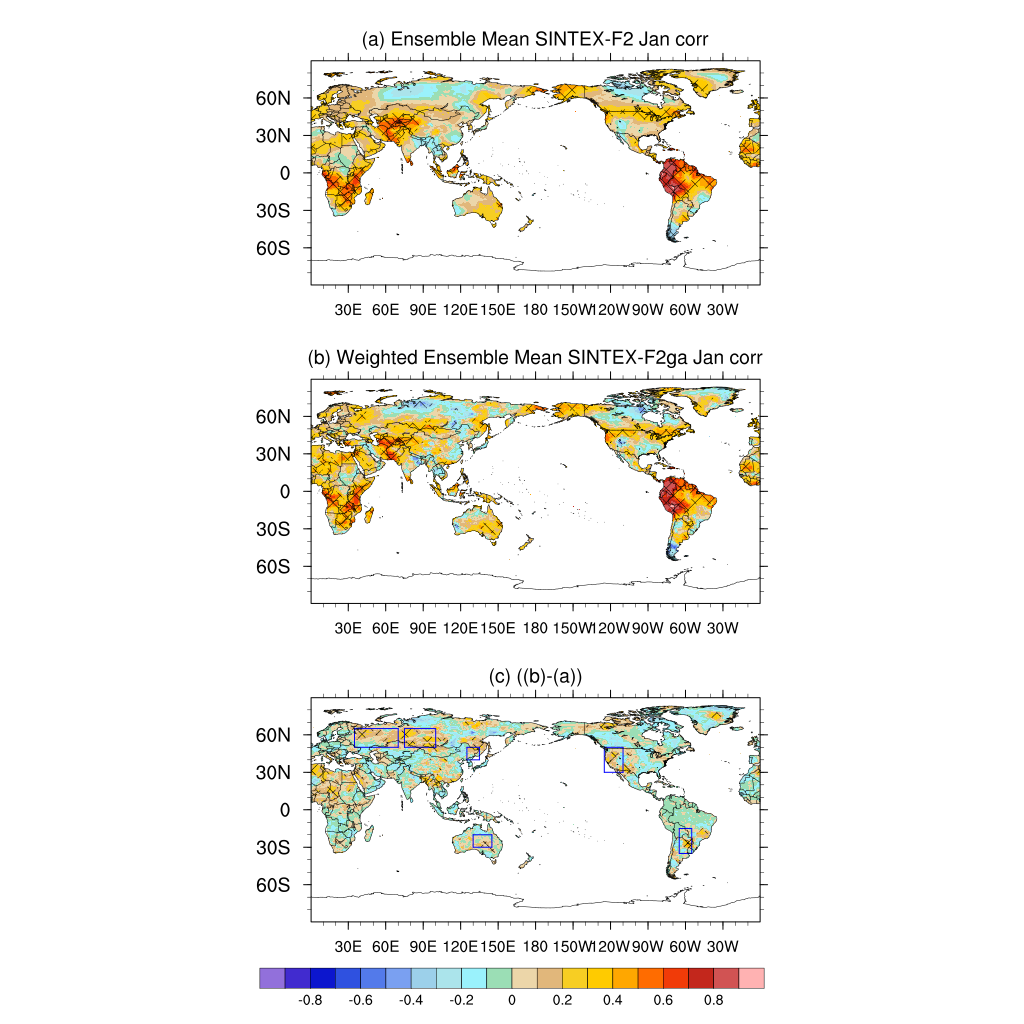


Fig S21. Same as Fig S20 but for Jan.

The figure was prepared using The NCAR Command Language (version 6.4.0) [Software]. (2017). Boulder, Colorado: UCAR/NCAR/CISL/TDD. <http://dx.doi.org/10.5065/D6WD3XH5>


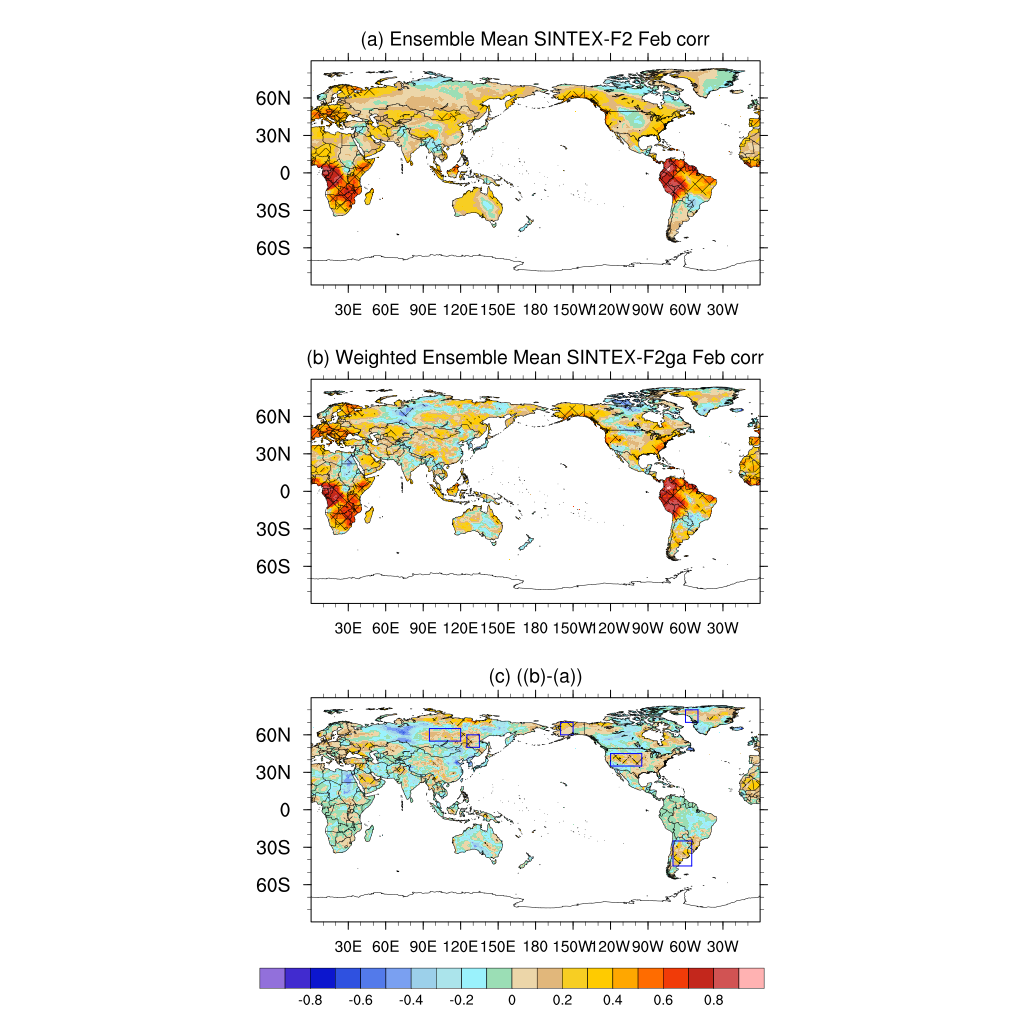


Fig S22. Same as Fig S20 but for Feb.

The figure was prepared using The NCAR Command Language (version 6.4.0) [Software]. (2017). Boulder, Colorado: UCAR/NCAR/CISL/TDD. <http://dx.doi.org/10.5065/D6WD3XH5>
